# Resource availability and capacity to implement multi-stranded cholera interventions in the north-east region of Nigeria

**DOI:** 10.1186/s44263-023-00008-3

**Published:** 2023-08-04

**Authors:** Kelly Elimian, Anwar Musah, Ozius Dewa, Carina King, Katerina Crawford, Emmanuel Pembi, Ifeanyi Ike, Puja Myles, Catherine Pritchard, Birger Carl Forsberg, Tobias Alfven

**Affiliations:** 1 Department of Global Public Health, Karolinska Institutet, Stockholm, Sweden; 2 Exhale Health Foundation, Abuja, Nigeria; 3 Department of Geography, University College London, London, UK; 4 School of Health Systems and Public Health, University of Pretoria, Pretoria, South Africa; 5 Adamawa State Ministry of Health, Yola, Adamawa State Nigeria; 6 Nigeria Centre for Disease Control, Abuja, Nigeria; 7 Clinical Practice Research Datalink, Medicines and Healthcare Products Regulatory Agency, London, UK; 8 Public Health Division, Nottinghamshire County Council, Nottingham, UK; 9 Sachs’ Children and Youth Hospital, Stockholm, Sweden

**Keywords:** Availability, Capacity, Cholera, Community engagement, Coordination, Nigeria, Surveillance, WASH

## Abstract

**Background:**

Limited healthcare facility (HCF) resources and capacity to implement multi-stranded cholera interventions (water, sanitation, and hygiene (WASH), surveillance, case management, and community engagement) can hinder the actualisation of the global strategic roadmap goals for cholera control, especially in settings made fragile by armed conflicts, such as the north-east region of Nigeria. Therefore, we aimed to assess HCF resource availability and capacity to implement these cholera interventions in Adamawa and Bauchi States in Nigeria as well as assess their coordination in both states and Abuja where national coordination of cholera is based.

**Methods:**

We conducted a cross-sectional survey using a face-to-face structured questionnaire to collect data on multi-stranded cholera interventions and their respective indicators in HCFs. We generated scores to describe the resource availability of each cholera intervention and categorised them as follows: 0–50 (low), 51–70 (moderate), 71–90 (high), and over 90 (excellent). Further, we defined an HCF with a high capacity to implement a cholera intervention as one with a score equal to or above the average intervention score.

**Results:**

One hundred and twenty HCFs (55 in Adamawa and 65 in Bauchi) were surveyed in March 2021, most of which were primary healthcare centres (83%; 99/120). In both states, resource availability for WASH indicators had high to excellent median scores; surveillance and community engagement indicators had low median scores. Median resource availability scores for case management indicators ranged from low to moderate. Coordination of cholera interventions in Adamawa State and Abuja was high but low in Bauchi State. Overall, HCF capacity to implement multi-stranded cholera interventions was high, though higher in Adamawa State than in Bauchi State.

**Conclusions:**

The study found a marked variation in HCF resource availability and capacity within locations and by cholera interventions and identified cholera interventions that should be prioritised for strengthening as surveillance and laboratory, case management, and community engagement. The findings support adopting a differential approach to strengthening cholera interventions for better preparedness and response to cholera outbreaks.

**Supplementary Information:**

The online version contains supplementary material available at 10.1186/s44263-023-00008-3.

## Background

Approximately 2.9 million cholera cases and 95,000 deaths occur annually in cholera-endemic countries [1], with most cases reported in sub-Saharan Africa [2]. Generally, case fatality rates (CFRs) associated with cholera from the African region are declining; however, some countries, including Nigeria, have reported increasing CFRs in recent years [3]. Most recently, amidst the COVID-19 pandemic, Nigeria recorded an unprecedented cholera outbreak, with over 93,000 cases and 3000 deaths (CFR of 3.5%) across 33 of its 36 States and the Federal Capital Territory between October 2020 and October 2021 [4]. The outbreak indicated the country’s increased vulnerability to cholera, especially in the northern region, which accounted for 90% of recorded cases [4].

Actualising the World Health Organization (WHO) Global Task Force on Cholera Control (GTFCC) strategic goals for cholera control—a 90% reduction in cholera deaths and cholera elimination in half of the cholera-endemic countries by 2030—relies on implementing six multi-stranded cholera interventions [5]. These interventions are (i) leadership and coordination, (ii) surveillance and laboratory, (iii) case management/healthcare system, (iv) oral cholera vaccine (OCV), (v) water sanitation and hygiene (WASH), and (vi) community engagement. The GTFCC advocates for the assessment of resource availability of each cholera intervention in order to identify gaps and inform appropriate planning and response in cholera-endemic countries [6]. For example, assessing a cholera-endemic country’s resource availability to implement surveillance and laboratory systems for cholera is crucial to early warning systems enhancement and evaluation of control efforts [7]. In 2018, the Nigeria Centre for Disease Control (NCDC)-led multisectoral Cholera Technical Working Group (TWG) reiterated the need for local assessment of the country’s resource availability and capacity to implement cholera multi-stranded interventions. The north-east region of the country with many known cholera hotspots (i.e. an area or a subpopulation exhibiting recurrent cholera cases, year after year) was considered a priority for such an assessment.

Nigeria is one of the four countries (others are Bangladesh, Namibia and the Democratic Republic of the Congo) to benefit from the GTFCC-led Cholera Support Platform. This global initiative for cholera control will require harnessing lessons learnt from previous cholera responses and, importantly, utilising context-specific evidence to revise the country’s National Strategic Plan of Action on Cholera Control. Understanding the country’s capacity to implement multi-stranded cholera interventions, particularly in healthcare facilities (HCFs), is critical to these endeavours [8]. However, our literature review and interactions with the NCDC-led cholera TWG at both state and national levels indicate a paucity of evidence on HCF resource availability to implement the multi-stranded cholera interventions in Nigeria. A study assessing the WHO African region’s readiness to prevent, detect, respond, and recover from cholera outbreaks found adequate preparation to prevent or control outbreaks; however, only nine West African countries (Benin, Burkina Faso, Cote d’Ivoire, Ghana, Mali, Niger, Senegal and Sierra Leone) participated in the study [9], excluding Nigeria with a substantial burden of the disease in the region [10]. Although the exact locations were not disclosed, a qualitative study on 193 Nigerian HCWs’ perspectives on the preparedness of HCFs for outbreaks of communicable diseases found that 98% of them perceived their facilities as insufficiently equipped to respond to disease outbreaks, with poor awareness of essential preparedness components, such as training, routine emergency drills, disease surveillance, and waste management [11].

Thus, to fill the gap in evidence, we aimed to assess HCF resource availability and capacity to implement multi-stranded cholera interventions in two North-Eastern states in Nigeria—Bauchi and Adamawa States— as well as the coordination of cholera interventions in the state and Abuja (where national coordination of cholera control takes place).

## Methods

### Study design

We conducted a cross-sectional survey assessing HCF resource availability and capacity to implement cholera interventions at the state level and coordination of cholera interventions at the state and national levels in Nigeria.

### Study theoretical and analytical framework

**Fig. 1:**
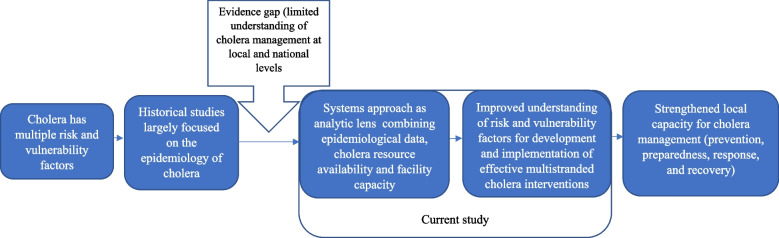
Systems’ conceptual framework to understanding the cholera management resource availability and capacity

Research on cholera has been predominantly epidemiological and primarily confined to the medical field with a reactive focus on outbreaks. Little information exists on HCF capacity and resource availability for implementing the multi-stranded cholera interventions and their effectiveness [12]. Considering the role played by disease surveillance systems, community engagements for awareness raising, health systems resilience, leadership, and coordination, among others, in cholera dynamics, an interdisciplinary approach should be adopted if the GTFCC strategic goal for 2030 is to be achieved. Therefore, we adopted a systems thinking approach [13] as a conceptual framework for this study, as it holds the promise to transcend the conceptual challenges noted above, in which studies on cholera are limited in their scope (see Fig. 1). Moreover, a systems approach becomes pertinent, given the need to place cholera multi-stranded interventions within the broader sustainable development discourse.

### Study setting

**Fig. 2:**
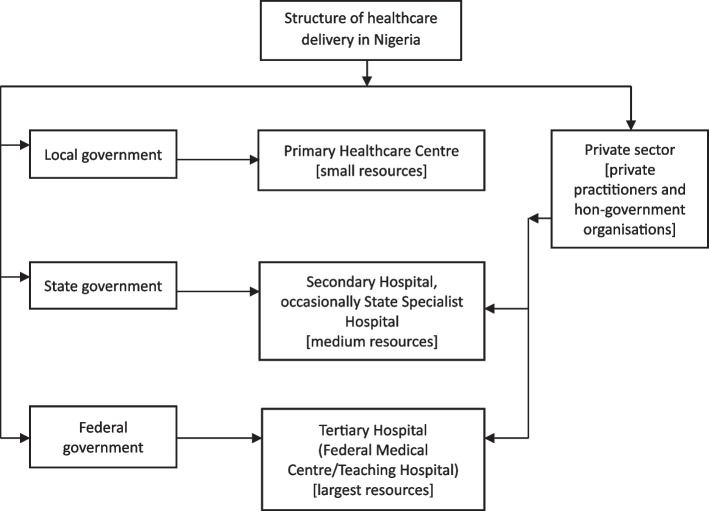
Schematic representation of healthcare delivery in Nigeria

Nigeria comprises 36 States and the Federal Capital Territory (Abuja). Healthcare delivery in Nigeria is a concurrent responsibility of the three tiers of government and the private sector [14], as depicted in Fig. 2.

This study was conducted in Adamawa and Bauchi States in the North-East region of Nigeria. Adamawa State has an estimated population of 4.7 million people across 21 local government areas (LGAs), while Bauchi State has about 7.5 million people across 20 LGAs [15]. Adamawa and Bauchi States were selected for this study because of cholera endemicity [16] and high levels of fragility with direct implications for increased cholera transmission. In 2018, Bauchi State recorded the highest number of cholera cases at 9405 and 35 deaths while Adamawa State recorded 2748 cholera cases and 41 deaths [16]. Furthermore, Bauchi State with 19,453 cholera cases (323 deaths) remained the Nigerian state with the highest absolute number of cholera cases between October 2020 and October 2021; however, Adamawa State with 754 cholera cases (32 deaths) recorded substantially lower number of cholera cases during this outbreak [4]. The states are considered fragile due, in part, to the high level of insurgency by Boko Haram and similar terrorist groups in the region. Insurgency is often characterised by severe development challenges including the destruction of social amenities (e.g. water pipes and health facilities) and displacement of people from their communities and source of livelihood [17]. It is also common for the government in this region, with support from NGOs, to establish camps for internally-displaced persons (IDP) in order to cater for their basic needs, including healthcare. In addition, we assessed the coordination of cholera interventions in Adamawa and Bauchi States as well as in Abuja (where the national coordination of cholera control is based).

### Sample size estimation and sampling

There was no formal sample size calculation for this study, considering pragmatic constraints dictated by the study setting (e.g. staff safety, travel and time). There are 760 HCFs (345 in Adamawa and 415 in Bauchi) considered functional during data collection. Within the LGAs considered safe by local partners, 120 HCFs were purposefully sampled based on managers formally expressing willingness to participate. Further, we ensured to select HCFs in contrasting settings (rural, peri-urban and urban LGAs) to account for the differential risk of cholera incidents by setting [16]. An urban LGA was classified by each State Epidemiologist using the criteria in an existing classification system in Nigeria [18].

### Data collection

Data collection was undertaken between 8 and 30 March 2021. Data on the coordination of cholera interventions were collected at two levels: state (Adamawa and Bauchi) and Abuja; HCF resource availability and capacity data were collected in Adamawa and Bauchi only. We developed the questionnaire by adapting three existing tools for assessing HCF service provision: the “WHO core questions and indicators for monitoring WASH in HCFs” [19], the “WHO guide for assessing cholera outbreak response and improving preparedness” [20], and the “WHO service availability and readiness assessment tool” [21]. Before data collection, we trained seven research assistants (including four LGA Disease Notification and Surveillance Officers) per state. Training included study-specific objectives and methodology, ethics and ethical considerations, the protocol for an Open Data Kit (ODK) Application [22] on password-protected mobile devices, and infection prevention and control (IPC).

The study questionnaire was arranged into three sections: “characteristics of HCF”, “assessment of cholera interventions”, and “assessment of cholera prevention and control coordination” (see the study questionnaire details in Additional file 1). The trained data collectors in each study area contacted the administrator or manager of a HCF to explain the study objectives and obtained permission for data collection. Following a HCF’s approval to undertake data collection, a data collector met with the facility manager to administer the questionnaire on an agreed date and time. Here, the data collector asked a question and read out the options for scoring. If data collection was postponed, data collectors rescheduled another visit to the HCF two times before continuing to the next HCF. While the trained data collectors were responsible for administering the first and second sections of the questionnaire to HCFs, the third section was administered by one of the researchers (KE) to cholera focal persons in the state and Abuja. The cholera TWG focal persons in each location had at least seven years of experience in cholera response.

### Data management

Data were downloaded from ODK and imported into Stata 16 (Stata Corp. LP, College Station, TX, USA) for management.

### Scoring of cholera interventions

#### Management of WASH, surveillance, and community engagement data

The scoring systems for WASH, surveillance, and community engagement are summarised in Additional files 2, 3 and 4. In summary, binary responses were given a score of ‘1’ if the questionnaire item was present (i.e. Yes) and ‘0’ if absent (i.e. No). Categorical responses were given an ordinal score, with a value of ‘2’ given the best option, ‘1’ for the next best option, and 0 if absent or the worst option (e.g. for ‘location of main water source’ was scored as follows: ‘2’ if the option is ‘on premises’, ‘1’ if ‘up to 500 m’, and ‘0’ if ‘500 m or further’). A similar scoring system was used to coordinate cholera interventions at the state and national levels. Resource availability scores for each cholera intervention’s indicators were generated based on the highest value available (see an excerpt in Table 1 and details in Additional files 2, 3 and 4).

**Table 1:** An excerpt of scores for WASH indicators

**Intervention**	**Indicator**	**Question**	**Scores for response options**	**Total possible score**
**WASH**	BWS^a^	Main water source	“1”: ‘Piped supply inside facility’, ‘piped supply outside health facility’, ‘tube well borehole’, and ‘protected dug well’ “0”: ‘No water source’, ‘rainwater from the roof’, and ‘tank ruck water vendor’	1
		Location of main water source	“2”: ‘On premises’ “1”: ‘Up to 500 m’ “0”: ‘500 m or further’	2
		Water availability from the main water source	“1”: ‘Yes’ “0”: ‘No’	1
		Interruption of main water source	“1”: ‘No’ “0”: ‘Yes’	1
		Availability of container/reservoir to conserve water for use	“1”: ‘Yes’ “0”: ‘No’	1
		Sufficiency of water quantity for health facility	“1”: ‘Yes’ “0”: ‘No’	1
				**Total = 7**

A similar scoring system was used for the remaining WASH indicators as well as surveillance and community engagement indicators

^a^BWS stands for basic water services

#### Management of case management data

In managing the case management variables, we acknowledged the duality and/or flexibility in the role of healthcare workers (see an excerpt in Table 2 and details in Additional file 3). In addition to their core duties, we recognised the fact that a nurse may be involved in fetching water within an HCF (a likely scenario in primary HCFs). Thus, a score of ‘1’ was given to multiple professions under ‘essential staff’ for cholera case management. For clinical staff, for example, we awarded a score of ‘1’ each for the presence of ‘medical doctor’ and ‘pharmacist’; however, a score of ‘1’ was awarded for the presence of ‘nurse’ or ‘medical ward helper’ or ‘community health extension worker’. The absence of any healthcare personnel was awarded a score of ‘0’. For questions without specific or quantifiable responses (e.g. what is the quantity of medical supply ‘x’ in your health facility), we used ‘10 cholera patients’ as a benchmark. The questions under each case management indicator were then recoded to get the total scores.

**Table 2:** An excerpt of scores for cholera case management and its respective indicators

**Indicator**	**Sub-indicator**	**Question**	**Scores for responses**	**Total score**
**Essential staff**	Clinical staff	-Medical doctor -Nurse/nurse helper -Medical ward helper -Stretcher/carrier -Pharmacist -Community health worker (CHW)	“1”: ‘Yes’ “0”: ‘No’	‘Medical doctor’: 1 ‘Pharmacist’: 1 ‘Nurse’ or ‘medical ward helper’ or ‘CHW’: 1
	IPC staff^a^	-WatSan officer -Cleaner -Laundry worker -Sprayer -Water carrier -Chlorinator/solution preparer -Watchman -Hygiene educator -Cook -Cook assistant	“1”: ‘Yes’ “0”: ‘No’	‘1’: ‘WatSan officer’ or ‘cleaner’ or ‘laundry worker’ or ‘sprayer’ or ‘water carrier’ or ‘chlorinator/solution preparer’ or ‘watchman’ or ‘hygiene educator’ ‘1’: ‘Cook’ or ‘cook assistant’
	Administrative staff	-CTC coordinator/supervisor for case management^b^ -Administrator for case management -Water, and sanitation supervisor -Logistics officer -Storekeeper	“1”: ‘Yes’ “0”: ‘No’	‘1’: ‘CTC coordinator/supervisor for case management’ or ‘administrator for case management’ ‘1’: ‘Logistics officer’ or ‘store-keeper’
				**Total = 7**

Scoring for the remaining case management indicators and their respective sub-indicators, with binary responses (present vs absent), are outlined in Additional file 3

^a^IPC stands for infection prevention and control

^b^CTC stands for cholera treatment centre

### Data analysis

Data analysis was conducted in three stages: (i) HCF level analysis including facility profiling, (ii) case-specific analysis to assess the scores for each intervention at the state and Abuja or national level, and (iii) comparative analysis to assess the differences in mean scores of the two states as well as performance for coordination in Abuja. Descriptive analyses and radar plots were performed using Stata. This paper was written following the Strengthening the Reporting of Observational Studies in Epidemiology (STROBE) checklist for cross-sectional studies [23] (see the completed checklist in Additional File 5).

#### Healthcare facility profiling

In describing the features of the HCFs surveyed, we presented continuous variables using median and inter-quartile range (IQR) as they were non-normally distributed; and categorical or binary variables were described using frequency and percentages (%).

#### Calculating the composite scores for cholera interventions and indicators

Using an approach previously used in Malawi [24], we created composite variables by grouping questions into indicators and indicators into respective cholera interventions (Table 3). Details of the analytical steps, as well as the composition of case management indicators and sub-indicators, are presented in Additional file 6.

**Table 3:** Categories of cholera interventions and their respective indicators

**Intervention**	**Indicator**	**Possible score**	**Total score**
**WASH**	Basic water services	7	36
	Basic sanitation services	10	
	Basic hygiene services	8	
	Basic healthcare waste management services	4	
	Basic environmental cleaning practices	7	
**Surveillance and laboratory**	Surveillance (epidemiology)	9	14
	Laboratory	5	
**Case management**	Availability of essential staff	3	71
	Availability of supplies for acute rehydration and ORS^a^	35	
	Availability of other medical commodities	13	
	IPC stewardship	17	
	Staff training	3	
**Community engagement**	Community engagement/risk communication	9	9
**Coordination**	Surveillance and laboratory	11	48
	Case management	9	
	WASH and oral cholera vaccination	8	
	Health systems	5	
	Leadership and coordination	9	
	Community engagement	6	

^a^ORS stands for oral rehydration solution

We plotted radar plots by calculating the average score of each indicator by the ‘State’ variable (Data from Adamawa and Bauchi was combined to provide a single overall score); the closer an indicator score is to 100, the better the indicator’s status, and vice-versa.

#### Determining resource availability for implementing cholera interventions

The availability of resources for implementing cholera interventions (first outcome variable) was derived by calculating an average percentage score based on the percentage scores of indicators. The variable was described using median and IQR, disaggregated by State. For ease of interpretation, the median scores were categorised as follows: low (0–50), moderate (51–70), high (70–90), and excellent (> 90). See additional details in Additional file 7.

#### Determining the adequacy of capacity for implementing cholera interventions

The capacity to implement cholera interventions (second outcome variable) was derived by recoding the average score (continuous variable) for each intervention indicator to binary: high (coded ‘1’) or low (coded ‘0’) capacity. Therefore, we defined high capacity as a score equal to or higher than the average indicator score, and low capacity as a score lower than the average indicator score.

## Results

### Description of the study health facilities

**Fig. 3:**
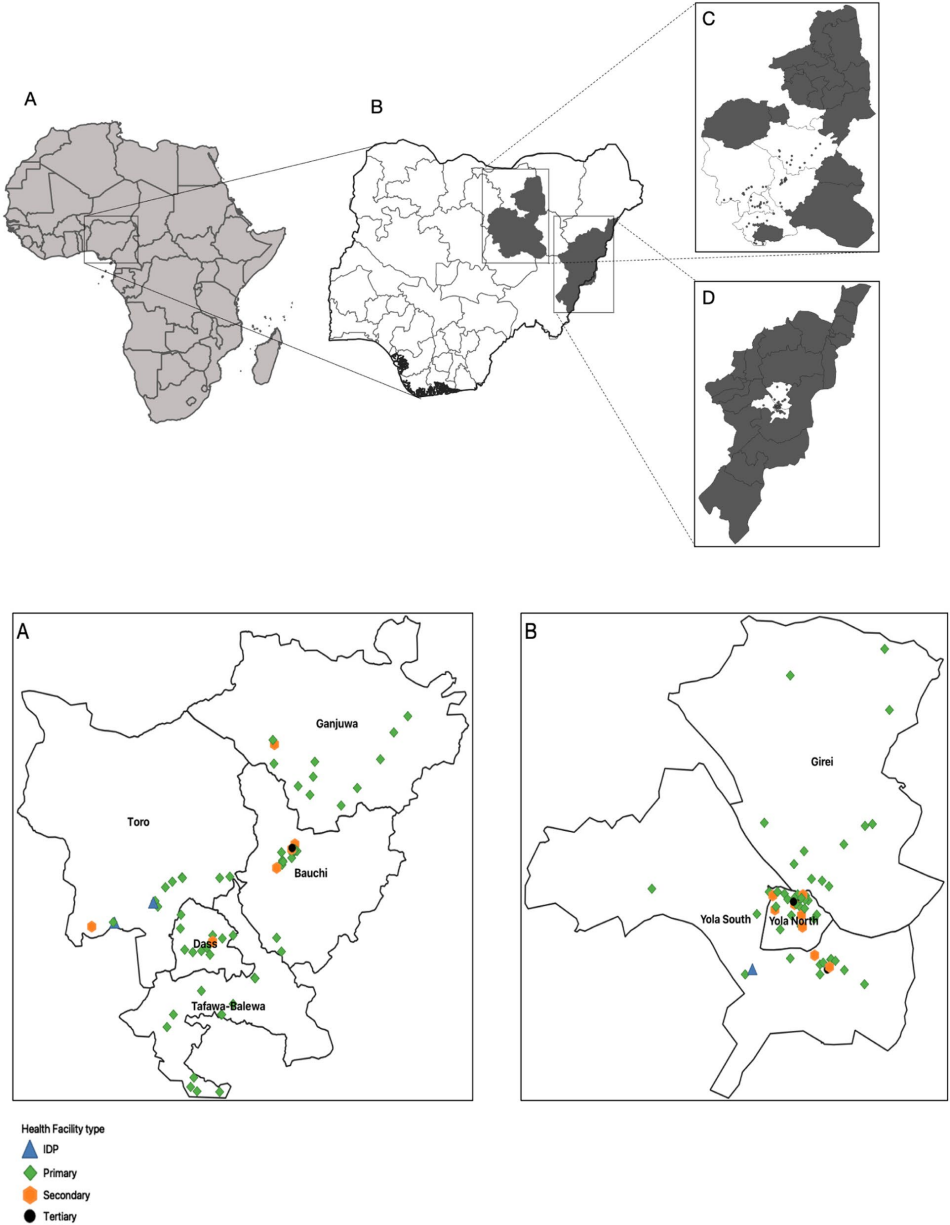
The study locations in Nigeria, including the spatial distribution of health facilities in Adamawa and Bauchi States; the upper part shows the map of Africa indicating the location of Nigeria (**A**), and the map of Nigeria (**B**) indicating the locations of Bauchi State (**C**) and Adamawa State (**D**); the lower part shows the distribution of health facilities in Bauchi State (**A**) and Adamawa State (**B**). IDP, internally displaced persons’ camp

The geospatial distribution of the 120 HCFs (55 in Adamawa State and 65 in Bauchi State) surveyed for the study is depicted in Fig. 3.

The majority (87%; 104/120) of the HCFs surveyed were in catchment areas classified as ‘cholera hotspots’ (Table 4). Most of the HCFs in both states were primary healthcare centres (78% in Adamawa State and 86% in Bauchi State) and government-owned (86% in Adamawa State and 88% in Bauchi State). While most HCFs in Adamawa State were in urban areas (76%), the majority in Bauchi State (66%) were in rural areas.

**Table 4:** Percentage distribution of surveyed facilities according to background characteristics

**Variable**	**Adamawa** **(*n* = 55)**	**Bauchi** **(*n* = 65)**	**Overall**^**a**^ **(*N* = 120)**
	**Frequency (%)**	**Frequency (%)**	**Frequency (%)**
**Estimated population in the catchment area of healthcare facility**			
< 5000	8 (15)	25 (39)	33 (28)
5000–10,000	11 (20)	20 (31)	31 (26)
10,001–20,000	24 (44)	10 (15)	34 (28)
> 20,000	12 (22)	10 (15)	22 (18)
**Healthcare facility catchment area is a cholera hotspot**			
No	13 (24)	3 (5)	16 (13)
Yes	42 (76)	62 (95)	104 (87)
**Healthcare facility operates 24 h**			
No	1 (2)	6 (9)	7 (6)
Yes	54 (98)	59 (91)	113 (94)
**Healthcare facility type**			
Primary	43 (78)	56 (86)	99 (83)
IDP	1 (2)	2 (3)	3 (3)
Secondary	8 (15)	6 (9)	14 (12)
Tertiary	3 (6)	1 (2)	4 (3)
**Healthcare facility ownership**			
Public	47 (86)	57 (88)	104 (87)
Private-for-profit	6 (11)	8 (1)	14 (12)
Private-for-non-profit (NGO or missionary)	2 (4)	0 (0)	2 (2)
**Healthcare facility setting**			
Rural	9 (16)	43 (66)	52 (43)
Urban	42 (76)	12 (19)	54 (45)
Peri-urban	4 (7)	10 (15)	14 (12)
**Capacity to admit patients for over 48 h**			
No	19 (35)	44 (68)	80 (67)
Yes	36 (66)	21 (32)	40 (33)
**Regular supply of electricity in healthcare facility**			
No	4 (7)	30 (46)	34 (28)
Yes	51 (93)	35 (54)	86 (72)
**Healthcare facility managed cholera cases in the past 5 years**			
No	31 (56)	26 (40)	57 (48)
Yes	24 (44)	39 (60)	63 (53)
**Healthcare facility with a dedicated CTU**			
No	48 (87)	65 (100)	113 (94)
Yes	7 (13)	0 (0)	7 (6)
**Is cholera treatment free?**			
No	2 (4)	5 (8)	7 (6)
Token taken	3 (6)	0 (0)	3 (3)
Yes	26 (47)	21 (32)	47 (39)
Unspecified	24 (44)	39 (60)	63 (53)
**Median (IQR) number of beds (excluding maternity) for admitting patients**^b^	7 (0–10)	12 (0–19)	8 (0–8)
**Median (IQR) number of patients hospitalised per day**	3.5 (3–7)	5 (3–16)	4.5 (3–10)
**Median (IQR) number of medical personnel**	10 (7–15)	11 (6–18)	10.5 (7–15)
**Median (IQR) number of non-medical personnel**	6 (4–10)	7 (4–9)	7 (4–9)

*CTU* cholera treatment unit

^a^Combined data from Adamawa and Bauchi States

^b^Only for admitting health facilities

### HCF resource availability to implement cholera interventions

**Fig. 4:**
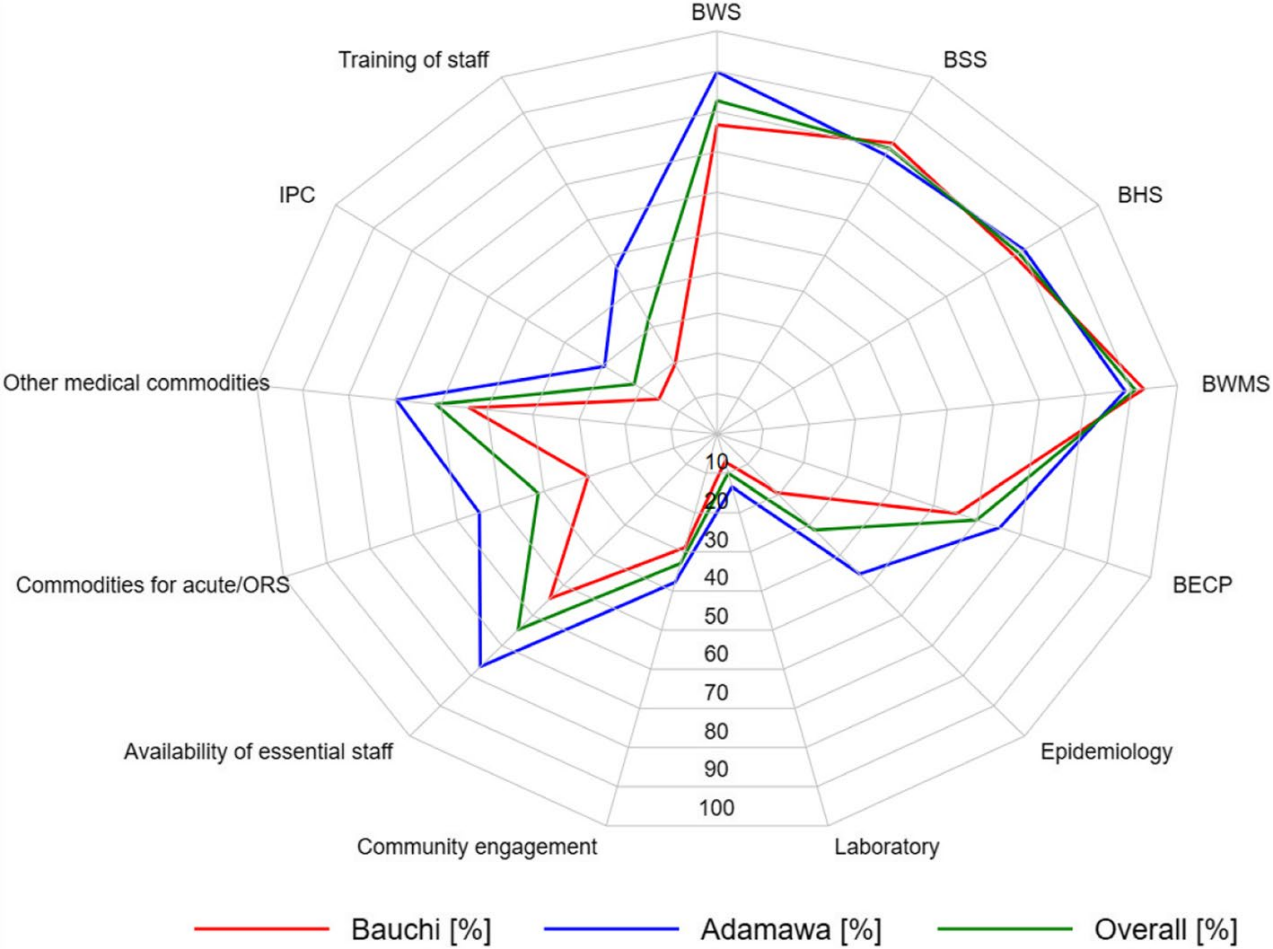
A radar plot showing resource availability of cholera interventions in Adamawa and Bauchi States, Nigeria. BWS, basic water services; BSS, basic sanitation services; BHS, basic hygiene services; BWMS, basic healthcare waste management services; BECP, basic environmental cleaning practices; IPC, infection prevention and control

Except for basic waste management and sanitation services, resources available for implementing cholera interventions were higher in Adamawa State (blue line of Fig. 4) than in Bauchi State (red line of Fig. 4). Overall (green line of Fig. 4), resources available for implementing WASH indicators were higher than those for the other cholera interventions. Resource availability median scores for the various cholera interventions and their respective indicators, presented in Additional file 7, are similar to the pattern observed in the radar plots (Fig. 4).

### HCF capacity to implement cholera interventions

In the overall population, HCFs can be classified into three categories (less than 50%, 50–59%, 60% and above) regarding their capacity to implement cholera interventions (Table 5). Less than 50% of the HCFs recorded high capacity for surveillance, laboratory, IPC stewardship, and community engagement. High capacity for basic healthcare waste management services, basic hygiene services, availability of supplies for acute rehydration and oral rehydration solution (ORS), availability of other medical commodities, and training of healthcare personnel were observed in 50–59% of the HCFs. All other interventions were recorded in 60% and above of the assessed HCFs. There were proportionally more HCFs demonstrating high capacity in Adamawa State compared to Bauchi State across almost all indicators, except for basic sanitation services and basic healthcare waste management services, in which Bauchi State performed better. Across all indicators scoring less than 50% of facilities with high capacity, Bauchi had the lowest proportions .

**Table 5:** Capacity of healthcare facilities to implement cholera interventions in Adamawa and Bauchi States, Nigeria

**Cholera intervention**	**Indicator**	**Adamawa** **(*n* = 55)**			**Bauchi** **(*n* = 65)**			**Overall** **(*N* = 120)**		
		**Average score**	**High capacity**	**Low capacity**	**Average score**	**High capacity**	**Low capacity**	**Average score**	**High capacity**	**Low capacity**
**WASH**	Basic water services	90	43 (78)	12 (22)	77	39 (60)	26 (40)	83	82 (68)	38 (32)
	Basic sanitation services	78	35 (64)	20 (36)	82	47 (72)	18 (28)	80	82 (68)	38 (32)
	Basic hygiene services	77	33 (60)	22 (40)	80	34 (52)	31 (48)	78	67 (56)	53 (44)
	Basic healthcare waste management services	89	39 (71)	16 (29)	93	47 (72)	18 (28)	91	86 (72)	34 (28)
	Basic environmental cleaning practices	65	29 (53)	26 (47)	55	32 (49)	33 (51)	60	61 (51)	59 (49)
**Surveillance and laboratory**	Surveillance	46	36 (66)	19 (35)	19	19 (29)	46 (71)	32	55 (46)	65 (54)
	Laboratory	14	29 (53)	26 (47)	7	10 (15)	55 (85)	10	39 (33)	81 (67)
**Case management**	Essential staff	77	48 (87)	7 (13)	54	32 (49)	33 (51)	65	80 (67)	40 (33)
	Supplies for acute rehydration and ORS	55	40 (73)	15 (27)	30	24 (37)	41 (63)	41	64 (53)	56 (47)
	Other medical commodities	70	33 (60)	22 (40)	54	30 (46)	35 (54)	61	63 (53)	57 (48)
	IPC stewardship	30	35 (64)	20 (36)	15	19 (29)	46 (71)	22	54 (45)	66 (55)
	Training of health personnel	47	40 (73)	15 (27)	20	25 (39)	40 (62)	32	65 (54)	55 (46)
**Community engagement**	Community engagement	38	27 (49)	28 (51)	29	21 (32)	44 (68)	33	48 (40)	72 (60)

High score was defined as a score equal to or higher than the average indicator score, while low score as a score lower than the average indicator score

### Coordination of cholera interventions at the state and national levels

**Fig. 5:**
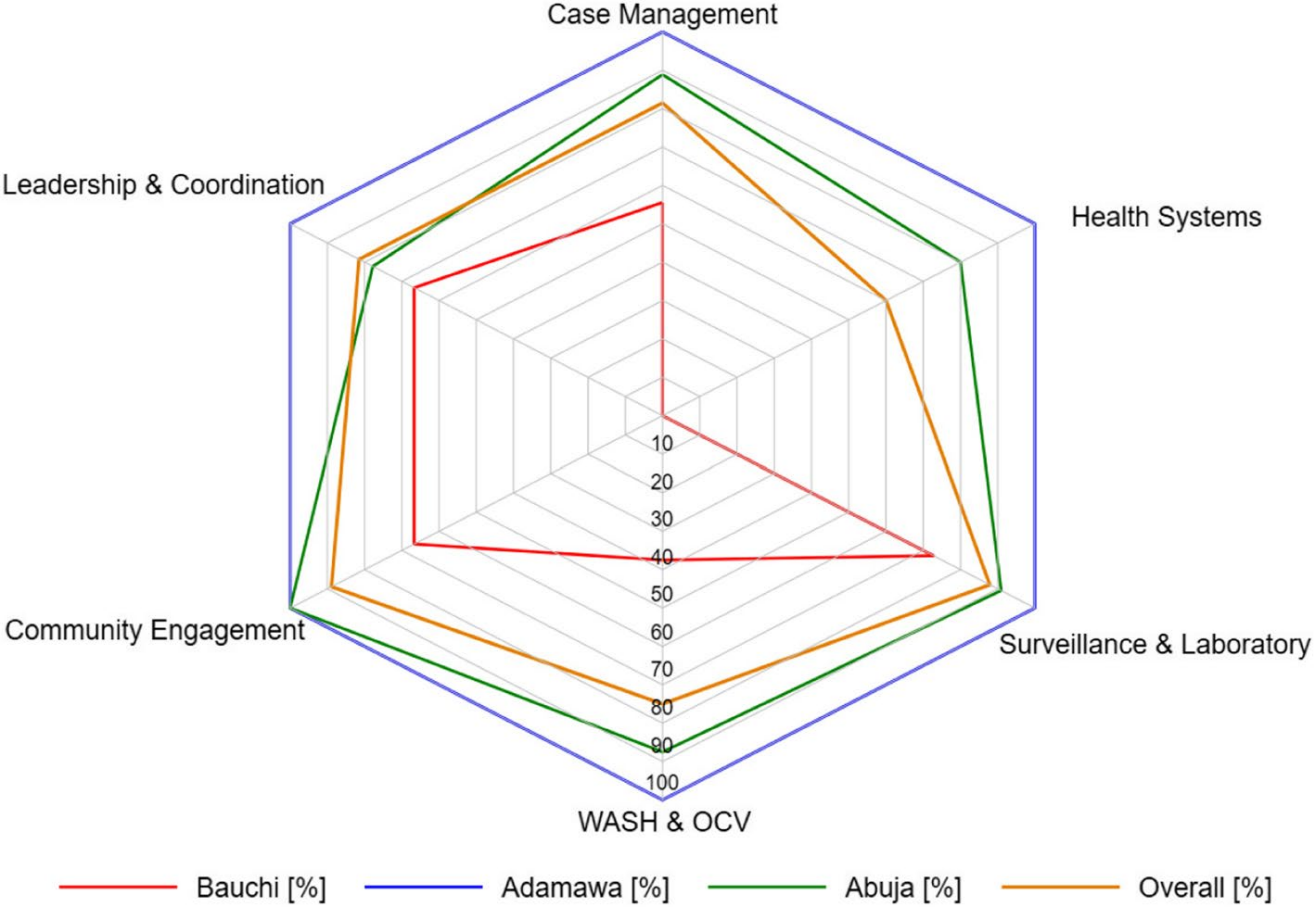
Radar plot showing the capacity for the coordination of cholera interventions in Adamawa, Bauchi, Abuja, and overall (a combination of the data from Adamawa and Bauchi). WASH, water, sanitation, and hygiene; OCV, oral cholera vaccination

Overall (orange line), coordination of surveillance/laboratory and community engagement was generally high, reaching up to 90% in the radar plot (Fig. 5). This was closely followed by case management, WASH/OCV, and leadership and coordination, with up to ≥ 80%. However, health system coordination was low at 60% overall. At the national level in Abuja (green line), coordination of all cholera interventions was high, especially community engagement, surveillance/laboratory, case management, and WASH/OCV, with ≥ 90% in the radar plot. Notably, coordination of all the cholera interventions was low in Bauchi State, especially that of the healthcare system at zero per cent (red line). However, the coordination of all the cholera interventions in Adamawa State was excellent at 100% (blue).

## Discussion

This study has provided the first comprehensive assessment of HCF resource availability and capacity to implement multi-stranded cholera interventions in a fragile and cholera-endemic region of Nigeria. This is particularly important for Nigeria which was not captured in the assessment of the African region’s readiness to prevent, detect, respond, and recover from a cholera outbreak [9], despite accounting for a substantial proportion of cholera cases and deaths in Africa [10]. Apart from Bauchi State, our findings suggest a robust HCF resource availability and capacity to implement multi-stranded cholera interventions for the prevention and control in Nigeria. Specifically, while we found the capacity for laboratory diagnosis of cholera to be low, particularly in Bauchi State, findings for the other African countries showed a different pattern, with laboratory indicators showing the best performance in eight countries [9]. The low laboratory capacity in our study locations could be attributable, in part, to the long-held practice of centralising laboratory diagnosis of clinical specimens from cholera reporting states at the Nigeria CDC central reference laboratory in Abuja, albeit rapid diagnostic tests are done at the state level. The inclusion of some countries (e.g. Madagascar) that had not reported any cholera cases for several years in the African regional assessment could partly explain the differences in findings. Nonetheless, cholera diagnosis in our study locations could readily be strengthened through substantial investments in the use of rapid diagnostic tests which performed very well in comparison to laboratory culture during the most recent cholera outbreak in Nigeria [4].

Our findings imply that effectively strengthening Nigeria’s capacity to respond to increasing cholera outbreaks will require both high capacity and resources to implement the multi-stranded interventions. However, considering resource constraints, the findings suggest that resource availability may be more crucial than capacity (which tends to improve with increasing experience from response to cholera outbreaks). This may explain why cholera interventions in Bauchi State (with lower capacity) which had managed more cases than Adamawa State (with higher capacity) in the past [16] and most recent [4] outbreaks in Nigeria need to be prioritised for strengthening. However, it seems that cholera endemicity and occurrence frequency (indicators of severity) had a reversed effect on HCF capacity and resource availability to implement cholera case management. This was exemplified by better resource availability and higher capacity for case management in Adamawa State than in Bauchi State, despite the higher burden of cholera in the latter than in the former state [4, 16]. However, we recommend caution in interpreting the finding, given that the analysed data were collected during the dry season. This is because HCFs in both locations tend to be more inclined to prepare for cholera response through the prepositioning of essential commodities during the rainy season (colloquially referred to as cholera season), which could potentially change the findings.

In the overall assessment, coordination of cholera interventions was very good at the national (Abuja) and sub-national levels in the present study. Paradoxically, surveillance and community engagement interventions which were weak in terms of availability in the HCF performed very well in terms of coordination across the three study locations (Adamawa, Bauchi, and Abuja). This mismatch between Abuja (i.e. national) and sub-national performance on coordination and the limited HCF coordination capacity is expected in emergency risk management. It is common for national public health authorities to have well-developed policies and legislative frameworks for governing outbreak response that is not matched with actual implementation (lack of action) at the sub-national or community levels [24]. Relative to other coordination indicators, health systems (availability of dedicated cholera TWG, specification of roles and responsibilities of TWG stakeholders, mobilisation and allocation of resources for preparedness, facilitation of simulation exercises and pre-positioning of essential supplies) performed poorly, with Bauchi scoring close to zero per cent under this indicator. This could be because health systems indicators captured elements of an outbreak or emergency preparedness in the early stages of development in most disaster risk management studies in the African region [25]. Perhaps because of its more rural nature, Bauchi State performed poorly across all coordination indicators compared to Abuja, Adamawa and overall assessment. This calls for the government and its stakeholders to provide coordination support to Bauchi State, considering the multisectoral nature of interventions to eliminate cholera which cannot be achieved without adequate coordination capacity.

Experiences from COVID-19 have highlighted the importance of the increased capacity for prediction, preparedness, and early response in controlling and reducing mortality [5]. Such capacity is strengthened if epidemiological and laboratory data are available to inform community and HCF preparedness for and mitigation against the adverse effects of cholera outbreaks. This study found inadequate capacity in these data-dependent areas which could partly explain the endemicity of cholera and the often devastating effects of cholera outbreaks recorded in the study areas, especially in Bauchi State [4]. The challenge of poor surveillance system for cholera in these areas extends to the request of OCV from GAVI through the GTFCC. Oftentimes, it derails the reactive mitigation of a severe cholera outbreak, as previously reported in Cameroon [26], and could negatively affect cholera case management, especially amidst other public health events, thereby resulting in high CFRs [4]. In the absence of an optimal community-based cholera surveillance system, which is currently the case in Nigeria, weak surveillance within the healthcare system can derail planning for risk communication activities (cholera risks per who, where, and when) and estimation of cholera burden, thus obscuring the country’s progress towards achieving the GTFCC’s global roadmap goals for cholera control. Moreover, Nigeria continues to rely on suspected case definitions for cholera surveillance with limited laboratory confirmatory tests in many areas. This was reiterated during the latest cholera outbreak in Nigeria when Adamawa State accounted for 17.6% of 329 laboratory-confirmed cholera cases and Bauchi State had no records of laboratory culture [4]. The problem with this approach is the dependence of specificity on cholera outbreak severity. During the cholera outbreak in Haiti in 2010, the cholera case definition had a sensitivity of 91% but a specificity of 43% [27]; however, the specificity was even lower (8%) in an African context, albeit retaining its high sensitivity (93%) [28]. Thus, it may not be ideal for Nigeria, where cholera is endemic, typically with periodic outbreaks every 3–4 years. Our findings, therefore, suggest that more attention should be given to strengthening routine data management systems to address the identified gap and inform evidence-based programming for effective cholera management in Nigeria, both in HCFs and communities.

In this study, staff training was considered a measure of resource availability for cholera case management. A report on the State of African Resilience posits that human capacity development is a central element for improved well-being as it provides the needed transformative capacity in the operationalisation of all other capacities required [29]. This is particularly crucial in a fragile region of the North-East where the human resource for health has been severely diminished through the outward migration of (especially non-indigenous) HCWs and the suspension of programmes providing external technical assistance [30]. This study found a gap in staff training, especially in Bauchi State, which can limit the capacity of HCFs to manage cholera cases effectively. Similar findings have been reported in Nigeria [11] and Cameroon [26]. The relatively better level of staff training in Adamawa compared to Bauchi could be explained by the preponderance of selected urban-based HCFs in the former (more prone to insurgency) and rural-based HCFs in the latter state (less prone to insurgency). This finding supports the prevailing analogy as to why HCWs in Nigeria tend to be reluctant to work in rural areas due to the poor work conditions, inadequate work resources, and limited social amenities [31]. The poor IPC stewardship in both study locations suggests an increased vulnerability of HCWs, patients, and relatives to hospital-acquired cholera infection, as reported previously during a cholera outbreak in northern Nigeria in 2010 [32].

Our findings suggest that the capacity of HCFs to implement cholera interventions is substantially sustained by more robust WASH/OCV services at the state levels. This is particularly important for the North-East region, where access to WASH services is abysmally low compared to the other country’s regions [33]. Notably, it was under this cholera intervention that Bauchi State outperformed Adamawa State particularly on basic sanitation services and basic healthcare waste management services. The relatively high performance of WASH/OCV indicators could be because cholera occurrence is significantly linked to poor access to water and sanitation services and the recommendation of OCV as an additional public health tool along with WASH for eliminating cholera. Evidence from the literature shows a significant growth in the broad adoption of WASH strategies in the fight against cholera among African countries [34], which became even more prominent during the response to the COVID-19 pandemic [35]. Additionally, regardless of location, we found better resource availability for WASH services in urban than in rural areas, which is per global trends [36]. Other than sanitation services, better WASH service in Adamawa State compared to that in Bauchi State is also in line with the national trends in Nigeria [33].

A study on cholera preparedness, response, and prevention in the Southern African Development Community highlighted that there is a robust socio-cultural discourse that exists concerning cholera [37]. This means that it is critical to consider community-held ideas, fears, and individual help-seeking behaviour regarding cholera in order to come up with appropriate and acceptable solutions [38]. Given that disasters occur at the community level where public health facilities are uniquely located, respectful community engagement in risk communication can play a crucial role in strengthening community preparedness and health system resilience [39] through its contribution to more accurate risk perception and improved level of awareness [40]. This is particularly important if there are mechanisms that allow the incorporation of local knowledge into the management of cholera outbreaks. We found the limited capacity to engage with communities among the participating HCFs. This finding is not peculiar to this study as a study in Uganda discovered the crucial role and benefits of community engagement and participation in disaster risk management intervention design and implementation [41]. However, a key question remains on how to consult and involve communities [41]. Leveraging lessons from recurrent cholera outbreaks in the North-East region of Nigeria [42], this could be addressed by dialoguing with communities respectfully and treating them as essential stakeholders (as opposed to just intervention recipients) in the design and implementation of intervention targeting cholera control [43].

To our knowledge, our study is the first comprehensive assessment of HCF resource availability and capacity to implement multi-stranded cholera interventions in a fragile and cholera-endemic setting. This is an essential step toward strengthening Nigeria’s capacity and possibly other cholera-endemic countries to actualise the GTFCC’s roadmap goals by 2030, as the evidence has provided context-specific gaps in the multi-stranded cholera interventions that should be prioritised for strengthening. Our study has also made methodological contributions of public health importance, particularly in the disaster risk management field. Often, countries endemic for cholera conduct self-assessments and report to international agencies (e.g. WHO and UNICEF) on the results of their preparedness and response to cholera. For example, Nigeria conducted the Joint External Evaluation (JEE) in 2017 and mid-term JEE in 2019 to assess its compliance with the International Health Regulation’s core capacities for prevention, detection, and response [44, 45]. However, being the first external and comprehensive assessment of capacity and resource availability for implementing cholera interventions in Nigeria, our study represents a departure from a self-assessment approach by addressing the potential for bias in reporting progress.

The study has some notable limitations. Firstly, without assessing all the HCFs in the study locations and a robust sample size estimation, our findings may be prone to selection bias and have limited generalisability, especially in Adamawa State with a preponderance of urban-based HCFs. Secondly, by generating composite variables for cholera interventions, we may have missed potentially helpful information on individual questions on cholera interventions; however, given the study’s objectives, pragmatically generating composite variables is ideal for organising multiple highly correlated variables into more digestible or meaningful information [46]. Thirdly, questions on the coordination of cholera interventions in the state and Abuja (national) were administered to a cholera focal person in each location. Without multiple opinions, especially of community members and HCF mangers who may have a different view of coordination mechanism, our findings may be prone to information bias. We did not assess the availability of resources for implementing oral cholera vaccination, a reflection of the common practice wherein state and national cholera working groups activate vaccination for reactive response to cholera outbreaks. Lastly, our definition of high capacity to implement multi-stranded cholera interventions was a relative concept, given that the average value of each cholera intervention depended on the overall performance of all the HCFs studied. This however highlights the need for an absolute set of criteria—clinical and policy relevance—for classifying HCF capacity within the cholera context.

## Conclusions

The study found a marked variation in HCF resource availability and capacity within locations and by cholera interventions, and identified cholera interventions that should be prioritised for strengthening as surveillance and laboratory, case management, and community engagement. The findings support adopting a differential approach to strengthening cholera interventions by local (e.g. the Nigeria CDC) and global (e.g. the Global Task Force on Cholera Control’s Cholera Support Platform) actors for better preparedness and response to cholera outbreaks. From a conceptual perspective, this study, by identifying interdisciplinary areas requiring interventions from the government and its stakeholders, has strengthened the argument for a systems-thinking approach (e.g. participatory group model building to identify leverage points for intervention) to addressing limited HCF resource availability and capacity to implement multi-stranded cholera interventions.

## Supplementary Information

**Additional file 1.** Data collection tool (questionnaire).

**Additional file 2.** Scores for WASH, surveillance, and community engagements.

**Additional file 3.** Scores for cholera case management and its respective indicators.

**Additional file 4.** Scores for the coordination of cholera interventions.

**Additional file 5.** Completed STROBE checklist for cross-sectional studies.

**Additional file 6.** Calculation of composite scores for cholera interventions and their indicators.

**Additional file 7.** Resource availability median scores for cholera interventions and their respective indicators.

## Data Availability

Data are provided in the manuscript and its additional files.

## Abbreviations

HCF: Healthcare facility
WASH: Water, sanitation, and hygiene
CFR: Case fatality rate
WHO: World Health Organization
GTFCC: Global Task Force on Cholera Control
OCV: Oral cholera vaccine
NCDC: Nigeria Centre for Disease Control
TWG: Technical Working Group
NGO: Non-governmental organisation
LGA: Local Government Area
ODK: Open Data Kit
IPC: Infection prevention and control
STROBE: Strengthening the Reporting of Observational Studies in Epidemiology
IQR: Inter-quartile range
JEE: Joint External Evaluation

## References

[1] Ali M, Lopez AL, You Y, Kim Y, Sah B, Maskery B, Clemens J. The global burden of cholera. Bull World Health Organ. 2012;90:209–18. 22461716 10.2471/BLT.11.093427 PMC3314202

[2] Perez-Saez J, Lessler J, Lee EC, Luquero FJ, Malembaka EB, Finger F, Langa JP, Yennan S, Zaitchik B, Azman AS. The seasonality of cholera in sub-Saharan Africa: a statistical modelling study. Lancet Glob Health. 2022;10:e831–9. 35461521 10.1016/S2214-109X(22)00007-9 PMC9090905

[3] Cholera Platform. Cholera outbreaks in Central and West Africa: 2020 Regional Update-Week 1–53. 2020.

[4] Elimian K, Yennan S, Musah A, et al. Epidemiology, diagnostics and factors associated with mortality during a cholera epidemic in Nigeria, October 2020-October 2021: a retrospective analysis of national surveillance data. BMJ Open. 2022;12:e063703. 36123095 10.1136/bmjopen-2022-063703 PMC9486350

[5] Global Task Force on Cholera Control. Ending cholera: a global roadmap to 2030. 2017. p. 32.

[6] Global Task Force on Cholera Control. Framework for the development and monitoring of a multisectoral national cholera plan: June 2019. Geneva: 2019.

[7] Global Task Force on Cholera Control (GTFCC) Surveillance Working Group (2017) Interim guidance document on cholera surveillance. Annecy.

[8] Lee CT, Buissonnière M, McClelland A, Frieden TR. Association between preparedness and response measures and COVID-19 incidence and mortality. 2021. medRxiv 2021.02.02.21251013.

[9] Sodjinou VD, Keita M, Chamla D, et al. Assessment of the countries’ readiness to detect and control cholera outbreaks in the WHO African Region. Arch Clin Biomed Res. 2022;06:656–62.

[10] Sodjinou VD, Talisuna A, Braka F, et al. The 2021 cholera outbreak in West Africa: epidemiology and public health implications. Arch Clin Biomed Res. 2022;6:296–307.

[11] Ughasoro MD, Esangbedo DO, Udorah IM. Health-care workers’ perspectives on preparedness of health-care facilities for outbreak of communicable diseases in Nigeria: a qualitative study. Am J Trop Med Hyg. 2019;100:1022. 30652657 10.4269/ajtmh.18-0404 PMC6447122

[12] Lessler J, Moore SM, Luquero FJ, et al. Mapping the burden of cholera in sub-Saharan Africa and implications for control: an analysis of data across geographical scales. Lancet. 2018;391:1908–15. 29502905 10.1016/S0140-6736(17)33050-7 PMC5946088

[13] Arnold RD, Wade JP. A definition of systems thinking: a systems approach. Procedia Comput Sci. 2015;44:669–78.

[14] Adeyemo DO. Local government and health care delivery in Nigeria: a case study. J Hum Ecol. 2005;18:149–60.

[15] Nigeria Data Portal. Nigeria population by age and sex. 2020. Nigeria population by age and sex. https://nigeria.opendataforafrica.org/htmbyze/nigeria-population-by-age-and-sex. Accessed 1 Jun 2020.

[16] Elimian KO, Musah A, Mezue S, et al. Descriptive epidemiology of cholera outbreak in Nigeria, January–November, 2018: implications for the global roadmap strategy. BMC Public Health. 2019;19:1–11. 31519163 10.1186/s12889-019-7559-6 PMC6743111

[17] Diaconu K, Falconer J, Vidal N, O’May F, Azasi E, Elimian K, Bou-Orm I, Sarb C, Witter S, Ager A. Understanding fragility: implications for global health research and practice. Health Policy Plan. 2020;35:235–43. 31821487 10.1093/heapol/czz142 PMC7050687

[18] Ofem BI. A review of the criteria for defining urban areas in Nigeria. J Hum Ecol. 2012;37:167–71.

[19] World Health Organization and the United Nations Children’s Fund (UNICEF). Core questions and indicators for monitoring WASH in health care facilities in the Sustainable Development Goals. Geneva; 2018.

[20] World Health Organization. Cholera outbreak: assessing the outbreak response and improving preparedness. Geneva; 2004.

[21] World Health Organization. Service Availability and Readiness Assessment (SARA): an annual monitoring system for service delivery. Geneva; 2015.

[22] ODK (2022) ODK - Collect data anywhere. https://getodk.org/. Accessed 10 Oct 2022.

[23] Cuschieri S. The STROBE guidelines. Saudi J Anaesth. 2019;13:S31. 30930717 10.4103/sja.SJA_543_18 PMC6398292

[24] Dewa O, Makoka D, Ayo-Yusuf OA. Assessing capacity and implementation status of the disaster risk management strategy for health and community disaster resilience in Malawi. Int J Disaster Risk Sci. 2021;12:673–88.

[25] Ayenew T, Tassew SF, Workneh BS. Level of emergency and disaster preparedness of public hospitals in Northwest Ethiopia: a cross-sectional study. Afr J Emerg Med. 2022;12:246–51. 35795819 10.1016/j.afjem.2022.05.007 PMC9249593

[26] Ateudjieu J, Yakum MN, Goura AP, et al. Health facility preparedness for cholera outbreak response in four cholera-prone districts in Cameroon: a cross sectional study. BMC Health Serv Res. 2019;19:458. 31286934 10.1186/s12913-019-4315-7 PMC6615310

[27] Lucien MAB, Schaad N, Steenland MW, Mintz ED, Emmanuel R, Freeman N, Boncy J, Adrien P, Joseph GA, Katz MA. Identifying the most sensitive and specific sign and symptom combinations for cholera: results from an analysis of laboratory-based surveillance data from Haiti, 2012–2013. Am J Trop Med Hyg. 2015;92:758–64. 25732682 10.4269/ajtmh.14-0429 PMC4385769

[28] Nadri J, Sauvageot D, Njanpop-Lafourcade B-M, et al. Sensitivity, specificity, and public-health utility of clinical case definitions based on the signs and symptoms of cholera in Africa. Am J Trop Med Hyg. 2018;98:1021–30. 29488455 10.4269/ajtmh.16-0523 PMC5928804

[29] Cooke JG. The state of African resilience: understanding dimensions of vulnerability and adaptation. 2015.

[30] Ager AK, Lembani M, Mohammed A, Mohammed Ashir G, Abdulwahab A, de Pinho H, Delobelle P, Zarowsky C. Health service resilience in Yobe state, Nigeria in the context of the Boko Haram insurgency: a systems dynamics analysis using group model building. Confl Health. 2015;9:30. 26442129 10.1186/s13031-015-0056-3 PMC4593224

[31] Nwankwo ON, Ugwu C, Nwankwo G, Akpoke MA, Anyigor C, Obi-Nwankwo U, Andrew S, Nwogu K, Spicer N. A qualitative inquiry of rural-urban inequalities in the distribution and retention of healthcare workers in southern Nigeria. PLoS One. 2022;17:e0266159. 35349602 10.1371/journal.pone.0266159 PMC8963562

[32] Oladele DA, Oyedeji KS, Niemogha M-TT, et al. An assessment of the emergency response among health workers involved in the 2010 cholera outbreak in northern Nigeria. J Infect Public Health. 2012;5:346–53. 23164563 10.1016/j.jiph.2012.06.004 PMC7102686

[33] Federal Ministry of Water Resources, Government of Nigeria, National Bureau of Statistics (NBS), UNICEF. Water, sanitation and hygiene: national outcome routine mapping (WASH NORM) 2019: a report of findings. Abuja: FCT; 2020.

[34] D’Mello-Guyett L, Gallandat K, Van Den Bergh R, Taylor D, Bulit G, Legros D, Maes P, Checchi F, Cumming O. Prevention and control of cholera with household and community water, sanitation and hygiene (WASH) interventions: a scoping review of current international guidelines. PLoS One. 2020;15:e0226549. 31914164 10.1371/journal.pone.0226549 PMC6948749

[35] Dan-Nwafor C, Ochu CL, Elimian K, et al. Nigeria’s public health response to the COVID-19 pandemic: January to May 2020. J Glob Health. 2020. 10.7189/jogh.10.020399. 33274062 10.7189/jogh.10.020399 PMC7696244

[36] United Nations Children’s Fund (UNICEF) and World Health Organization (WHO). Progress on drinking water, sanitation and hygiene in Africa 2000–2020: five years into the SDGs. New York; 2022.

[37] Said MD, Funke N, Jacobs I, Steyn M, Nienaber S. The case of cholera preparedness, response and prevention in the SADC region: a need for proactive and multi-level communication and co-ordination. Water SA. 2011;37:559–66.

[38] Schaetti C, Sundaram N, Merten S, Ali SM, Nyambedha EO, Lapika B, Chaignat CL, Hutubessy R, Weiss MG. Comparing sociocultural features of cholera in three endemic African settings. BMC Med. 2013. 10.1186/1741-7015-11-206. 24047241 10.1186/1741-7015-11-206 PMC4016292

[39] Ryan B, Johnston KA, Taylor M, McAndrew R. Community engagement for disaster preparedness: a systematic literature review. Int J Disaster Risk Reduct. 2020;49:101655.

[40] Adame BJ. The persuasive efficacy of real versus salient hazard scenarios in motivating citizen-level hazard preparedness. Int J Disaster Risk Reduct. 2018;31:292–301.

[41] Neema S, Bua GM, Tuhebwe D, Ssentongo J, Tumuhamye N, Mayega RW, Fishkin J, Atuyambe LM, Bazeyo W. Community perspective on policy options for resettlement management: a case study of risk reduction in Bududa, Eastern Uganda. PLoS Curr. 2018. 10.1371/CURRENTS.DIS.49E8E547DE25CA1C1F9EDBBFC8B9EFA5. 10.1371/currents.dis.49e8e547de25ca1c1f9edbbfc8b9efa5 PMC6100022 30191081

[42] Ngwa MC, Wondimagegnehu A, Okudo I, et al. The multi-sectorial emergency response to a cholera outbreak in internally displaced persons camps in Borno state, Nigeria, 2017. BMJ Glob Health. 2020;5:2000. 10.1136/bmjgh-2019-002000 PMC7042583 32133173

[43] Barker KM, Ling EJ, Fallah M, Vandebogert B, Kodl Y, Macauley RJ, Viswanath K, Kruk ME. Community engagement for health system resilience: evidence from Liberia’s Ebola epidemic. Health Policy Plan. 2020;35:416–23. 32040166 10.1093/heapol/czz174

[44] Nigeria Centre for Disease Control. Country-led midterm joint external evaluation of IHR core capacities. Abuja; 2020.

[45] World Health Organization. Joint external evaluation of IHR core capacities of the Federal Republic of Nigeria. Geneva; 2017.

[46] Song MK, Lin FC, Ward SE, Fine JP. Composite variables: when and how. Nurs Res. 2013;62:45. 23114795 10.1097/NNR.0b013e3182741948 PMC5459482

