# Supplementary material for: Resource availability and capacity to implement multi-stranded cholera interventions in the north-east region of Nigeria
Source: BMC Glob Public Health. 2023 Aug 4;1:6. doi: 10.1186/s44263-023-00008-3 (PMC11622880; doi:10.1186/s44263-023-00008-3)
Supplement: Supplementary file 1 — Additional file 1. Data collection tool (questionnaire). [file 44263_2023_8_MOESM1_ESM.docx]

**Additional file 1: Data collection tools**

# Characteristics of health facility

| **Health facility characteristics** | |
| --- | --- |
| **Variable** | **Comment** |
| Date: |  |
| State: Adamawa or Bauchi |  |
| Local government area: |  |
| Is the local government area a cholera hotspot? | *Using existing classifications from hotspot mapping* |
| Estimated population of catchment area: |  |
| Position of interviewee: |  |
| Year of experience of interviewee: |  |
| Is the facility opened 24h/7day?   - No - Yes |  |
| Facility type   - Primary (public) - Secondary (public) - Tertiary (public) - Camp for internally-displaced persons |  |
| Facility ownership type   - Public - Private-for-profit - NGO - Mission hospital - Other (specify) |  |
| Health facility setting   - Rural - Peri-urban - Urban |  |
| Average walking distance for most patients to the health facility (in hours)?   - <1h - 1-5hrs - >5hrs |  |
| What is the average number of consultation in outpatient unit (excluding vaccination, antenatal care, and growth monitoring) per day? |  |
| Does the facility have the capacity to hospitalise/admit patients for ≥48 hours?   - Yes - No |  |
| If yes to QX, what is the number of beds (excluding maternity) for admitting patients? |  |
| If yes to QX, what is the average number of patient hospitalised per day? | *Only if applicable* |
| Is there a regular supply of electricity the health facility?   - No - Yes |  |
| If yes to QX1, what is the primary source of electricity for the health facility?   - Power Holding Company of Nigeria - Generator - Solar/Inverter - Other (specify): |  |
| If yes to QX1, how many hours of power in the last 24 hours? | *Specify hours* |
| Has the health facility managed cholera patients in the past 5 years?   - Yes - No |  |
| If not, why? | *Response in free-texts* |
| Does the health facility have a dedicated cholera treatment centre (CTC/CTU)?   - No - Yes |  |
| Was the CTC/CTU available during the last cholera outbreak?   - No - Yes |  |
| How many cholera-specific beds are available in the CTC/CTU? |  |
| Is cholera treatment free of charge?   - No - Token is collected - Yes |  |
| If not, how much money is usually charged for each patient? |  |
| Is the health facility fenced?   - No - Yes - Partially (incomplete fencing) |  |
| Is there a guard at entrance/exit of the health facility?   - No - Yes |  |
| **Availability of health personnel (medical + non-medical) and training** | |
| What is the total number of medical healthcare workers at the health facility? |  |
| What is the total number of non-medical healthcare workers at the health facility? |  |

# WaSH

| **Questions** | **Comment** |
| --- | --- |
| **Basic water services** | |
| G-W1. What is the main water supply for the facility? (Tick one)   - Piped supply inside the building (if yes, skip to G-W3) - Piped supply outside the building - Tube well / Borehole - Protected dug well - Unprotected dug well - Protected spring - Unprotected spring - Rain water catchment from roof - Tanker truck/Water vendor - Surface water (river/dam/lake/pond) - Other (specify) ____________________ - Don’t know (skip to G-S1) - No water source (skip to G-S1) | For each option, tick yes/no, and for yes: observed/not observed  *Note: If there is more than one source, the one used most frequently should be selected. If patients need to bring water from home because water is not available at the facility, “no water source” should be selected.* |
| G-W2. Where is the main water supply for the facility located?   - On premises - Up to 500 m - 500 m or further | *Note: On premises means within the building or facility*  *grounds.*  *This question refers to the location from where the water is accessed for use in the health facility (e.g. tap, borehole), rather than the source where it originates.* |
| G-W3. Is water available from the main water supply at the time of the survey?   - Yes - No   If yes, state the method used to confirm availability:   - Observation - Health worker’s report | *Note: To be considered available, water should be available at the facility at the time of the survey.* |
| Are there any times during the year when you have to find alternate source of water for the health facility?   - Yes - No |  |
| If “Yes”, please specify when?   - Dry season - Wet season |  |
| What is the frequency of interruption of the main source of water in the last 1 year (in days)? | *If less than one day, write zero ‘0’* |
| What is the typical duration of water interruption in the last 1 year (in days)? | *If less than one day, write zero ‘0’* |
| What is the longest duration of interruption ever experienced in the last 1 year (in days)? | *If less than one day, write zero ‘0’* |
| Do you have containers (reservoirs) to conserve water for use?   - No - Yes |  |
| Total volume of all these containers in litres | *If no container, write zero ‘0’* |
| Do you judge sufficient the quantity of water available for activities?   - Yes - No | *5–400 litres/person/day is the current standard* |
| **Basic sanitation services** | |
| G-S1. What type of toilets/latrines are at the facility for patients?   - Flush /Pour-flush toilet to sewer connection - Flush /Pour-flush toilet to tank or pit - Pit latrine with slab - Composting toilet - Flush /Pour-flush toilet to open drain - Pit latrine without slab/open pit - Bucket - Hanging toilet/latrine - Bush - Lake/river - No toilet/latrine (skip to G-H1) - Other (specify): ___________ | *Note: If more than one type of toilet is used, the most common type of toilet/latrine in the service area should be selected.* |
| G-S2. Is at least one toilet usable (available, functional, private)?   - Yes - No | *Note: To be considered usable, a toilet should be available, functional and private at the time of the survey or questionnaire.* |
| G-S 3-5. Are there toilets that …   - 3. Are in sex-separated or gender-neutral rooms? - 4. Do female toilets have capacity to manage menstrual hygiene? - 5. Are accessible for people with limited mobility? | *Responses to questions: yes/no/not sure* |
| Are toilets separated for staff and patients?   - Yes - No |  |
| Is the number of latrine/toilet sufficient?   - No - Yes | *1 toilet for every 20 users for inpatient setting; at least 4 toilets per outpatient setting; separate toilets for patients and staff* |
| Are soap and water currently available at toilets?   - Yes, within 5 m of toilets - Yes, more than 5 m from toilets - No, no soap and/or no water |  |
| **Basic hygiene services** | |
| G-H1. Is there a functional hand hygiene facility (with soap or alcohol-based hand rubs) presently at points of care (treatment areas, waiting rooms and near latrines)?   - Yes - No, there are hand hygiene facilities at points of care but not functional, or lacking soap and water or alcohol-based hand rub. - No, no hand hygiene facilities at points of care - No, no hand hygiene facilities at the health care facility (if yes, skip to G-C1) | *Note: For facilities with multiple consultation rooms or areas, select one at random and observe if a functional hand hygiene facility is present. A functional hand hygiene facility is any device that enables staff, patients and visitors to clean their hands effectively. It may consist of soap and water with a basin/pan for washing hands, or alcohol-based hand rub (ABHR). If ABHR is used, health care staff may carry a dispenser around between points of care. Chlorinated water (a prepared solution of chlorine suspended in water) is not considered an adequate substitute for soap and water or for ABHR.*  *Points of care are any location in the health care facility where care or treatment is delivered (e.g. consultation/exam rooms).*  *The term “hand hygiene” is used in place of “handwashing”, because this is an umbrella term that also includes cleaning hands with ABHR.* |
| G-H2. Is there a functional handwashing facility presently at one or more toilets?   - Yes - No, there are handwashing facilities near the toilets but lacking soap and/or water - No, no handwashing facilities near toilets (within 5 meters) | *Note: Handwashing facilities at toilets must include water and soap, rather than ABHR alone, since ABHR does not remove faecal matter. Check “yes” if at least one toilet has a handwashing facility with soap and water within 5 meters.* |
| If any handwashing facility was indicated in the last question, kindly specify the type (e.g. tippy taps): |  |
| Are soap and water (or alcohol-based hand rub) currently available in consultation rooms?   - Yes - Partially (e.g. lacking materials) - No |  |
| Do you have presently have disinfectants and latex gloves for IPC?   - Yes - No - Not sure |  |
| Is there an IPC focal person at the health facility?   - No - Yes - Not sure |  |
| **Basic health care waste management services** | |
| G-WM1. Is waste correctly segregated into at least three labelled bins in the consultation area?   - Yes, waste is segregated into three labelled bins - No, bins are present but do not meet all requirements or waste is not correctly segregated - No, bins are not present | *Note*  *For facilities with multiple consultation rooms, select one at random and observe whether sharps waste, infectious waste and non-infectious general waste are segregated into three different bins.*  *The bins should be colour-coded and/or clearly labelled, no more than three quarters (75%) full, and each bin should not contain waste other than that corresponding to its label. Bins should be appropriate to the type of waste they are to contain; sharps containers should be puncture-proof and others should be leak-proof. Bins for sharps waste and infectious waste should have lids.* |
| G-WM2. How does this facility usually treat/ dispose of infectious waste?   - Autoclaved - Incinerated (two chamber, 850-1000 °C incinerator) - Incinerated (other) - Burning in a protected pit - Not treated, but buried in lined, protected pit - Not treated, but collected for medical waste disposal off-site - Open dumping without treatment - Open burning - Not treated and added to general waste - Other (specify) | *Note*  *If more than one applies, select the method used most often.*  *Methods considered to meet the basic service level include autoclaving; incineration; burial in a lined, protected pit; and collection for medical waste disposal off-site.* |
| G-WM3. How does this facility usually treat/ dispose of sharps waste?   - Autoclaved - Incinerated (two chamber, 850-1000 °C incinerator) - Incinerated (other) - Burning in a protected pit - Not treated, but buried in lined, protected pit - Not treated, but collected for medical waste disposal off-site - Open dumping without treatment - Open burning - Not treated and added to general waste - Other (specify) | *Note*  *If more than one applies, select the method used most often.*  *Methods considered to meet the basic service level include autoclaving; incineration; burial in a lined, protected pit; and collection for medical waste disposal off-site.* |
| **Basic environmental cleaning practices** | |
| G-C1. Are cleaning protocols available?   - Yes - No | *Note*  *Protocols should include:*  *• step-by-step techniques for specific tasks, such as cleaning a floor, cleaning a sink, cleaning a spillage of blood or body fluids, and*  *• a cleaning roster or schedule specifying responsibility for cleaning tasks and frequency at which they should be performed.*  *The term for protocols may differ according to local practice; they may be referred to as Standard Operating Procedures (SOPs), guidelines, instructions, etc.*  *Where possible, protocols should be observed by the enumerator.* |
| G-C2. Have all staff responsible for cleaning received training?   - Yes, all have been trained - No, some but not all have been trained - No, none have been trained - No, there are no staff responsible for cleaning | *Note*  *“Staff responsible for cleaning” refers to non-health care providers such as cleaners, orderlies or auxiliary staff, as well as health care providers who, in addition to their clinical and patient care duties, perform cleaning tasks as part of their role.*  *Training refers to structured training plans or programs led by a trainer or appropriately qualified supervisor.* |
| At what frequency are the toilets cleaned?   - 1x/day - 2x/day - Only when it is dirty |  |
| Only if applicable: In a maternity waiting room, do the women/mothers help clean the toilets?   - No (staff do the cleaning) - Yes |  |
| Are protocols for cleaning the health facility (floor, sink, stool, spillage of blood or bodily fluid) and cleaning schedule available?   - Yes - No - Don’t know |  |
| Is there a trained WaSH/IPC focal person at your health facility?   - No - Yes |  |
| Does the health facility have a budget line for WaSH?   - Yes - No |  |
| Do you face difficulties obtaining the necessary supplies for WaSH?   - Yes - No - Sometimes | Ask to a cleaner |
| Frequency of cleaning the work environment (with soap and water or appropriately diluted disinfectant) during the survey? | Observe and state the hours of observation |

# Surveillance and laboratory

| **Question** | **Comment** |
| --- | --- |
| **Surveillance** |  |
| Do you have staff that trained in rapid response to cholera outbreak?   - No - Yes |  |
| If yes, how many staff have been trained in rapid response in the last 5 years?  Specify: |  |
| How many staff can perform epidemiological reporting of cholera (i.e. line listing)?  Specify: |  |
| Do you have staff trained in contact tracing of cholera cases and their close contacts?   - No - Yes, but other diseases (e.g. Covid-19) - Yes |  |
| If yes, specify the number: |  |
| Do you have a cholera register at the health facility?   - Yes - No   If yes, sight for confirmation |  |
| Is there a cholera weekly reporting form at the health facility?   - Yes - No   If yes, sight for confirmation |  |
| Is there a case definition of suspected cholera on the wall (including signage for cholera symptoms)?   - No - Yes |  |
| Are there sufficient materials/guidelines to support field investigation missions by Rapid Response Team members?   - No - Yes |  |
| Does your health facility have internet to facilitate timely reporting of surveillance data to the relevant institutions (state epidemiology and NCDC)?   - No - Yes | *Not personal internet* |
| Does your health facility have an electronic/paper-based cholera surveillance database to aid the prompt recognition and notification of cholera outbreak?   - No - Yes |  |
| Are cholera surveillance activities (trends and thresholds for alert and regular analysis etc.) carried out regularly (e.g. every 3 months)?   - No - Yes |  |
| **Laboratory** |  |
| Is there a laboratory for cholera diagnosis in this health facility?   - No - Yes |  |
| If not, where is laboratory diagnosis of cholera performed?  Specify: |  |
| If you answered yes to the question above, do you have staff able to diagnose cholera?   - No - Yes |  |
| If yes, specify the diagnostic method(s)  Options: culture, RDT, both |  |
| How many staff are trained in the laboratory diagnosis of cholera?  Specify: |  |
| Are there rapid diagnostic kits available in your health facility?   - Yes - No   Confirm if the response is ‘Yes’ |  |
| If yes, are the kits still valid (i.e. within the expiration date)?   - Yes - No |  |
| Do you have staff trained on collection, transportation and disposal of cholera samples?   - Yes - No |  |
| If yes, how many?  Specify: |  |
| Does the health facility have transport media for cholera diagnosis?   - Yes - No |  |
| If yes, specify the transport media:   - Filter paper - Cary Blair Medium - Locally developed medium - >1 medium |  |
| If any transport media is selected, do you consider the quantity available to your health facility to be of sufficient quantity?   - No - Somewhat - Yes |  |
| Are you able to carry out antimicrobial susceptibility test to aid cholera case management?   - No - Yes |  |
| Do you have the molecular capacity to perform *Vibrio cholerae* strain identification?   - Yes - No |  |

# Case management/healthcare system

| Are these essential health workers available for case management?   - CTC coordinator/supervisor - Administrator - Medical doctor - Nurses and/or nurse helpers - Medical ward helper - Stretcher-carrier - Pharmacist - Logistics, Water and Sanitation supervisor - Logistic officer - Store keeper - Cook - Cook-assistant - WatSan officer - Cleaner - Laundry worker - Sprayer-watchman - Water carrier - Chlorinator/solution preparer - Home visitor/Community health worker - Watchman/ sprayer - Hygiene educator   *Ask for each personnel’s availability (dual role is acceptable); response: yes/no* |
| --- |
| Availability [quantity] of medical supplies  First response: Yes/no; for Yes, ask: on average, *how many*   - Cuff for blood pressure - Stethoscope - Thermometers + disinfectant - Pair of scissors - Register - Sprayer at entrance, containing a chlorine solution 0.2% - Buckets of 20L with cover for chlorine solutions 2% and 0.2% - Sprayers - Drum 125L for washing hands (containing 0.05% chlorine solution) - Soap - Broom, floor cloth, dustbin with cover - Table, chair - Mats - Pairs of rubber gloves - Bucket of 20L with tap for ORS - Bucket of 20L with tap for drinkable water - Bucket of 10L (separate for stool and for vomit) - Cups, plates, spoons - Patients follow up forms - Rope: for hanging infusion bags and medical files - Hooks - Note book for transmission (shifts) - Pens, permanent marker - Oral rehydration salt - IV catheter (for adults and children) - Syringe (disposable) - Rubber boots - Pierced bed - Blanket - Loincloth - Tray, bottle, Kocher, small dish (cupule), kidney dish - Containers for dirty needles - Box examination gloves for single use - Infusion sets - Needles - Naso gastric tubes - Syringes 60ml feeding (Luer and conical tip) - Roll cotton wool (500g) - Gauze bandages - Adhesive tape - Bottle of polyvidone iodine 10% (200 ml) - 200L Ringer lactate - 5L Dextrose 5% - Vials Glucose hypertonic (50%) - Ampoule Furosemide (10mg/ml) - Ampoule Diazepam (5mg/ml) - Ampoule Quinine Di-Hydrochloride (300mg/ml) - Tablets of Doxycycline (100mg) - Tablets of Artesunate (50mg) - Tablets of Sulfadoxine Pyrimethamine (if no resistance) - Tablets of Acetyl Salicylic Acid (500mg) - Tablets of Paracetamol (500mg) - Bags of ORS |
| Availability of a separate kitchen for cooking   - Yes - No |
| Neutral area and kitchen   - Food store, shelves, table, chair, register, pens - Uniforms: one per worker - Cooking-pots of 50L - ladles - Fuel - Cups, plates, spoons - Drums of 125L (0.05% chlorine solution) for hand washing - Showers - Bucket of 20L with a tap in each shower - Sprayer for insecticide for the whole centre |
| Supplies for an ORP   - Bucket with lid and cup (or tap) for handwashing - Jug of 1 litre with lid to prepare ORS - NaDCC (0.5mg) tablets to prepare portable water for ORS - ORS sachets |
| Does health facility have ORT corners for the treatment of mild/moderate cholera cases?   - No - Yes |
| Is there a national protocol for cholera case management?   - Yes - No |
| Estimated number of cholera cases managed in the last 1 year: |
| Do you have staff trained in use of PPE for cholera case management?   - Yes - No |
| - Cholera Treatment Centres (CTC) and Units (CTU) are hospital structures where severe patients are isolated and receive specialized care including IV rehydration. CTCs are placed at central level while CTUs are smaller, decentralized inpatient facilities. - Oral rehydration points (ORP) are simple structures that provide oral rehydration to moderate cases. They must be decentralised and widespread in order to provide early rehydration for moderate cases and to identify severe cases for quick referral. - CTC and CTU are hospital structures. They must function 24 hours; ORP can be open 12 hours/day. |

# Community engagement

| Are there staff specifically tasked with community mobilisation/sensitisation on cholera prevention and control?   - No - Yes |
| --- |
| Are there community representatives in your disease outbreak preparedness committee?   - Yes - No |
| Does health facility organise cholera awareness sessions in community gatherings (e.g. schools and religious centres)?   - No - Yes, sometimes - Yes, regularly |
| Does health facility regularly engage community leaders and religious leaders to convey key public health messages in preparedness for an outbreak?   - No - Yes |
| Does health facility often engage community volunteers to promote health awareness?   - Yes - No |
| Do you communicate with community members via social media platforms (e.g. WhatsApp and Facebook) to provide health updates?   - No - Yes |
| What’re the 3 most common methods through which you reach out to community members?  Specify: |
| Do you practice community-based surveillance?   - Yes - No |
| If yes, provide a brief description: |
| Is there a mechanism for monitoring and enforcing food safety and water quality standards in your locality?   - No - Yes |

# Coordination and leadership

| **Question** | **Comment** |
| --- | --- |
| **Coordination of case management** | |
| Does a human-resources plan for cholera outbreak response exist**?**  If yes, is it based on defined competencies? | *Possible responses:*   - *Yes* - *Partly* - *No* |
| Do procedures exist for integrating volunteers into case management during a cholera outbreak? | Configure ODK to have a dropdown list for state vs Abuja |
| Do needs assessments determine the frequency and content of case management training, as well as the number of trainees? |  |
| Have sufficient resources been allocated for case management training at the **national/state** level? |  |
| Are supplies for cholera case management periodically tested, and are expired or inappropriate items disposed of in accordance with established guidelines? |  |
| Are cholera case management supplies (including PPE) determined on the basis of risk assessments and analyses? |  |
| Is there a system in place for managing severe cholera cases in the community (i.e. at the scene)? |  |
| Is a standardized cholera triage system in place in the event of a surge? |  |
| Are there guidelines and procedures for the establishment of CTC/CTU/ORP during a cholera outbreak? |  |
| **Surveillance** | |
| Do cholera TWG leads at the national/state level have access to historical surveillance data to inform planning? | *Endeavour to verify all positive responses* |
| Does surveillance system have protocols defining roles, responsibilities and procedures for the standardisation, collection, management, analysis and dissemination of data? |  |
| Does surveillance system provide for data-sharing with other cholera stakeholders outside the ministry of health/health facility (e.g. primary healthcare development agency, ministries of environment, water resources, and education)? |  |
| Do mechanisms exist for carrying out rapid training of health workers for cholera outbreak response?  Are the necessary resources and trained staff available for doing so? |  |
| Are essential laboratory supplies and equipment for cholera diagnosis determined on the basis of risk analyses? |  |
| Are essential laboratory supplies and equipment for cholera diagnosis readily available in sufficient quantities? |  |
| Are laboratory supplies periodically tested, and are expired products disposed of in accordance with established guidelines? |  |
| Do procedures exist for the exceptional procurement of laboratory supplies? |  |
| Are the triggers/criteria for switching from routine cholera surveillance to outbreak surveillance defined? |  |
| Are there mechanisms in place to establish a flexible cholera surveillance system that operates in IDP camps? |  |
| **Healthcare system** | |
| Has your state adopted the National strategic plan of action on cholera control? | An adaptation of the GTFCC global roadmap strategy |
| Does your state have a dedicated cholera TWG that can easily be activated into an EOC during an outbreak? | *Endeavour to verify all positive responses* |
| if yes, is the cholera TWG funded and functional? |  |
| Does the TWG specify the roles and responsibilities of all stakeholders? |  |
| Does the cholera TWG at the national/state level mobilise and allocate resources for cholera preparedness (pre-positioning)? |  |
| Does the cholera TWG at the national/state level facilitate simulation exercise on cholera outbreak response?  If it does, how often in a year? |  |
| Does your health facility practice ‘pre-positioning’ to ensure essential supplies are available at the beginning of an outbreak? |  |
| **WASH** | |
| Does the cholera TWG at the national/state level work with the ministries of environment, water resources, health and agriculture? | *Endeavour to verify all positive responses* |
| Are there mechanisms in place to ensure adequate WASH services for displaced populations? |  |
| **Oral cholera vaccination** | |
| Are the specific roles of OCV stakeholders (e.g. primary healthcare development agency, ministries of health and water resources) clearly defined? | *Endeavour to verify all positive responses* |
| Are OCVs periodically tested, and are expired or inappropriate vaccines disposed of in accordance with established guidelines? |  |
| Is there a system in place, including cold chain, for the distribution of OCVs and equipment in the event of an outbreak? |  |
| Do procedures exist for the exceptional request and delivery of OCVs response to a cholera outbreak? |  |
| If yes, are the procedures efficient? |  |
| Are there logistics (e.g. cold chain) in place for the implementation of OCV in your state/Nigeria? |  |
| Are there mechanisms in place to ensure the availability of OCVs for health care workers and vulnerable population (e.g. IDP camp residents)? |  |
| **Community and stakeholder engagement/risk communication** | |
| Are the responsibilities of pillars/health workers related to cholera risk communication defined? | *Endeavour to verify all positive responses* |
| Are the reports on cholera TWG published and disseminated regularly? |  |
| Do cholera risk communication strategies also target minority and vulnerable populations, such as persons residing in IDP camps? |  |
| Do coordination mechanisms exist to ensure the consistency of information supplied to the public? |  |
| Do procedures exist for the communication of risk information by community members to public health institutions? |  |
| Are the triggers or criteria for switching from routine to outbreak risk information communication defined? |  |
| **Leadership and coordination** | |
| Does the state/national cholera TWG have any regulations guiding the entry of foreign/local health partners to provide relief services during an outbreak? | *Endeavour to verify all positive responses* |
| Are there regulations guiding donations of health and medical supplies for cholera response? |  |
| Are resources (staff, supplies, finances) and systems (emergency- operations centres, transport and communications systems) sufficient to allow the cholera TWG fulfil its mandate? |  |
| Are funds available for multisectoral preparedness for and response to cholera outbreak at the national/state level? |  |
| Are multisectoral financing procedures available for the request, acceptance and utilisation of international financial assistance (or other resources) for cholera outbreak response? |  |
| Is there a national/state budget for preparedness and response to cholera control/outbreak? |  |
| Do mechanisms exist for the rapid mobilisation of additional resources (personnel, equipment and materials) to and between states (e.g. from Jigawa to Bauchi) during a cholera outbreak? |  |
| Do mechanisms for hospital networking during a cholera outbreak exist? |  |
| Is there a logistics system in place at the state/national level that includes tracking, monitoring and reporting components? |  |
| **Other** | |
| What are the 3 most important barriers to cholera coordination at the national/state level? | *Open ended response required* |
| What are the 3 most important enabling factors of cholera coordination at the national/state level? |  |
| How would you describe the participation of government in campaigns/measures aimed at controlling/eliminating cholera in Nigeria/your state in the last 5 years? |  |
| What are the 3 most important cholera interventions to achieve the GTFCC’s goals in your state/Nigeria?   - Community engagement - Healthcare system strengthening - Surveillance and reporting - Water, sanitation and hygiene - Oral cholera vaccine - Leadership and coordination |  |
