# Supplementary material for: Resource availability and capacity to implement multi-stranded cholera interventions in the north-east region of Nigeria
Source: BMC Glob Public Health. 2023 Aug 4;1:6. doi: 10.1186/s44263-023-00008-3 (PMC11622880; doi:10.1186/s44263-023-00008-3)
Supplement: Supplementary file 2 — Additional file 2. Scores for WASH, surveillance, and community engagements. [file 44263_2023_8_MOESM2_ESM.docx]

**Additional file 2: Scores for WASH, surveillance, and community engagements**

| **Intervention** | **Indicator** | **Question** | **Scores for response options** | **Total possible score** |
| --- | --- | --- | --- | --- |
| **WASH** | BWS | Main water source | “1”: ‘Piped supply inside facility’, ‘piped supply outside health facility’, ‘tube well borehole’, and ‘protected dug well’  “0”: ‘No water source’, ‘rain water from roof’, and ‘tank ruck water vendor’ | 1 |
|  |  | Location of main water source | “2”: ‘On premises’  “1”: ‘Up to 500 metre’  “0”: ‘500 metre or further’ | 2 |
|  |  | Water availability from main water source | “1”: ‘Yes’  “0”: ‘No’ | 1 |
|  |  | Interruption of main water source ‡ | “1”: ‘No’  “0”: ‘Yes’ | 1 |
|  |  | Availability of container/reservoir to conserve water for use | “1”: ‘Yes’  “0”: ‘No’ | 1 |
|  |  | Sufficiency of water quantity for health facility | “1”: ‘Yes’  “0”: ‘No’ | 1 |
|  |  |  | | **Total=7** |
|  | BSS | Type of toilet/latrine at health facility | “2”: ‘Flush/pour flush toilet to sewer connection’, ‘flush/poor flush toilet to tank or pit’, and ‘flush/pour flush toilet to open drain’  “1”: ‘Pit latrine with slab’  “0”: ‘No toilet/latrine’ | 2 |
|  |  | At least one toilet is usable, available, functional, and private | “1”: ‘Yes’  “0”: ‘No’ | 1 |
|  |  | Toilet is sex separate or gender neutral | “1”: ‘Yes’  “0”: ‘No’ | 1 |
|  |  | Toilet allows females to manage menstrual hygiene | “1”: ‘Yes’  “0”: ‘No’ | 1 |
|  |  | Toilet is accessible to persons with limited mobility | “1”: ‘Yes’  “0”: ‘No’ | 1 |
|  |  | Toilets are separate for staff and patients | “1”: ‘Yes’  “0”: ‘No’ | 1 |
|  |  | Sufficiency of the number of toilet/latrine | “1”: ‘Yes’  “0”: ‘No’ | 1 |
|  |  | Availability of soap and water at toilets | “2”: ‘Yes, within 5m of toilet’  “1”: ‘Yes, but >5m from toilet’  “0”: ‘No soap and/or water’ | 2 |
|  |  |  | | **Total=10** |
|  | BHS | Availability of functional hand hygiene facility at points of care | “2”: ‘Yes, available and functional’  “1”: ‘No, hand hygiene facility at point of care but not functional’  “0”: ‘No, no hand hygiene facility at health facility’, or ‘No, no hand hygiene facility at point of care’ | 2 |
|  |  | Availability of functional handwashing facility at one or more toilets | “2”: ‘Yes, available and functional’  “1”: ‘Handwashing facility near toilet but lacks soap &/or water’  “0”: ‘No handwashing facility near toilet (within 5m)’ | 2 |
|  |  | Availability of soap/water or alcohol-based hand rub in consultation | “2”: ‘Yes’  “1”: ‘Partially’  “0”: ‘No’ | 2 |
|  |  | Availability of disinfectants and latex gloves for IPC | “1”: ‘Yes’  “0”: ‘No’ | 1 |
|  |  | Availability of IPC focal person in health facility | “1”: ‘Yes’  “0”: ‘No’ | 1 |
|  |  |  | | **Total=8** |
|  | BWMS | Correct segregation of wastes into at least 3 labelled bins in consultation room | “2”: ‘Yes, waste is segregated into three labelled bins’  “1”: ‘No, bins are present but do not meet all requirements/waste is not correctly segregated’  “0”: ‘No, bins are not present’ | 2 |
|  |  | Method of medical waste disposal | “1”: ‘Burning in a protected pit’, or ‘contracted to a company/individual’, or ‘incinerated (two chamber, 850-1000 °C incinerator)’, or ‘not treated, but buried in lined, protected pit’, or ‘not treated, but collected for medical waste disposal off-site’, or ‘other incineration’  “0”: ‘Open burning’, or ‘not treated and added to general waste’ | 1 |
|  |  | Method of sharp medical waste disposal | “1”: ‘Burning in a protected pit’, or ‘contracted to a company/individual’, or ‘incinerated (two chamber, 850-1000 °C incinerator)’, or ‘not treated, but buried in lined, protected pit’, or ‘not treated, but collected for medical waste disposal off-site’, or ‘other incineration’  “0”: ‘Open burning’, or ‘not treated and added to general waste’ | 1 |
|  |  |  | | **Total=4** |
|  | BECP | Availability of cleaning protocol in health facility | “1”: ‘Yes’  “0”: ‘No’ | 1 |
|  |  | Training of all cleaning staff | “2”: ‘Yes, all have been trained’  “1”: ‘Some but not all have been trained’  “0”: ‘No, none has been trained’ | 2 |
|  |  | Frequency of toilet cleaning | “2”: ‘Twice a day’  “1”: ‘Once a day’  “0”: ‘Only when its dirty’ | 2 |
|  |  | Availability of protocols for health facility cleaning and cleaning schedule | “1”: ‘Yes’  “0”: ‘No’ | 1 |
|  |  | Availability of a trained WASH/IPC focal person in health facility | “1”: ‘Yes’  “0”: ‘No’ | 1 |
|  |  |  | | **Total=7** |
| **Surveillance** | Epidemiology | Staff training in rapid response to cholera outbreak | “1”: ‘Yes’  “0”: ‘No’ | 1 |
|  |  | Availability of sufficient materials/guidelines to support field investigation by rapid response team | “1”: ‘Yes’  “0”: ‘No’ | 1 |
|  |  | Staff training in cholera contact tracing | “1”: ‘Yes’  “0”: ‘No’ | 1 |
|  |  | Availability of cholera register | “1”: ‘Yes’  “0”: ‘No’ | 1 |
|  |  | Availability of cholera weekly reporting form | “1”: ‘Yes’  “0”: ‘No’ | 1 |
|  |  | Availability of cholera case definition/signage for symptoms on the wall | “1”: ‘Yes’  “0”: ‘No’ | 1 |
|  |  | Availability of internet for timely reporting of surveillance data | “1”: ‘Yes’  “0”: ‘No’ | 1 |
|  |  | Availability of electronic/paper-based cholera survey database for prompt recognition and notification of cholera outbreak | “1”: ‘Yes’  “0”: ‘No’ | 1 |
|  |  | Ongoing frequent cholera surveillance | “1”: ‘Yes’  “0”: ‘No’ | 1 |
|  |  |  | | **Total=9** |
|  | Laboratory | Availability of lab for cholera diagnosis | “1”: ‘Yes’  “0”: ‘No’ | 1 |
|  |  | Availability of RDT kits | “1”: ‘Yes’  “0”: ‘No’ | 1 |
|  |  | Availability of staff trained in the collection, transport, and disposal of stool sample for cholera diagnosis | “1”: ‘Yes’  “0”: ‘No’ | 1 |
|  |  | Capacity to perform antimicrobial resistance test | “1”: ‘Yes’  “0”: ‘No’ | 1 |
|  |  | Molecular capacity to perform *V. cholerae* strain identification | “1”: ‘Yes’  “0”: ‘No’ | 1 |
|  |  |  | | **Total=5** |
| **Community engagement** | Community engagement | Availability of staff specifically tasked with community mobilisation/sensitisation for cholera prevention and control | “1”: ‘Yes’  “0”: ‘No’ | 1 |
|  |  | Community representatives in cholera outbreak preparedness committee | “1”: ‘Yes’  “0”: ‘No’ | 1 |
|  |  | Health facility organises cholera awareness programmes at community gatherings (e.g., schools and religious centres) | “2”: ‘Yes, regularly’  “1”: ‘Yes, sometimes’  “0”: ‘No’ | 2 |
|  |  | Health facility regularly engage community leaders/religious to promote public health messages | “1”: ‘Yes’  “0”: ‘No’ | 1 |
|  |  | Health facility often engage community volunteers to promote health awareness | “1”: ‘Yes’  “0”: ‘No’ | 1 |
|  |  | Communication with community members via social media platforms to provide health updates | “1”: ‘Yes’  “0”: ‘No’ | 1 |
|  |  | Practice of community-based surveillance | “1”: ‘Yes’  “0”: ‘No’ | 1 |
|  |  | Availability of mechanism for monitoring/enforcing food safety and water quality | “1”: ‘Yes’  “0”: ‘No’ | 1 |
|  |  |  | | **Total=9** |
| ‡: Scoring system was flipped or reversed (e.g., ‘No’ was assigned a score of ‘1’ while ‘Yes’ a score of ‘0’) | | | | |
