# Supplementary material for: Resource availability and capacity to implement multi-stranded cholera interventions in the north-east region of Nigeria
Source: BMC Glob Public Health. 2023 Aug 4;1:6. doi: 10.1186/s44263-023-00008-3 (PMC11622880; doi:10.1186/s44263-023-00008-3)
Supplement: Supplementary file 3 — Additional file 3. Scores for cholera case management and its respective indicators. [file 44263_2023_8_MOESM3_ESM.docx]

**Additional file 3: Scores for cholera case management and its respective indicators**

| **Indicator** | **Sub-indicator** | **Question** | **Scores for responses** | **Total score** | |
| --- | --- | --- | --- | --- | --- |
| **Essential staff** | Clinical staff | Medical doctor  Nurse/nurse helper  Medical ward helper  Stretcher/carrier  Pharmacist  Community health worker | “1”: ‘Yes’  “0”: ‘No’ | ‘Medical doctor’: 1  ‘Pharmacist’: 1  ‘Nurse’ or ‘medical ward helper’ or ‘Community Health Extension Worker’: 1 | |
|  | IPC staff | -WatSan officer  -Cleaner  -Laundry worker  -Sprayer  -Water carrier  -Chlorinator/solution preparer  -Watchman  -Hygiene educator  -Cook  -Cook assistant | “1”: ‘Yes’  “0”: ‘No’ | ‘1’: ‘WatSan officer’ or ‘cleaner’ or ‘laundry worker’ or ‘sprayer’ or ‘water carrier’ or ‘chlorinator/solution preparer’ or ‘watchman’ or ‘hygiene educator’  ‘1’: ‘Cook’ or ‘cook assistant’ | |
|  | Administrative staff | -Cholera treatment centre coordinator/supervisor for case management  -Administrator for case management  -Water, and sanitation supervisor  -Logistics officer  -Store-keeper | “1”: ‘Yes’  “0”: ‘No’ | ‘1’: ‘CTC coordinator/supervisor for case management’ or ‘administrator for case management’  ‘1’: ‘Logistics officer’ or ‘store-keeper’ | |
|  |  | | | **Total=7** | |
| **Medical commodities** | Commodities for acute/rehydration care | -Bucket of 20L with tap for ORS  - Bucket of 20L with tap for drinkable water  -Rope for hanging infusion bags and medical files  -Hooks  -Oral rehydration salt  -IV catheter  -Syringe (disposable)  -Blanket  -Loincloth  -Tray, bottle, Kocher, small dish (cupule), kidney dish  -Containers for dirty needles  -Box of examination gloves for single use  -Infusion sets  -Needles  -Nasogastric tubes  -Roll cotton wool (500g)  -Gauze bandages  -Adhesive tape  -Bottle of polyvidone iodine 10% (200 ml)  -200L ringer lactate  -5L Dextrose (5%)  -Vials of glucose hypertonic (50%)  -Ampoule furosemide (10mg/ml)  -Ampoule diazepam (5mg/ml)  -Ampoule quinine di-hydrochloride (300mg/ml)  -Tablets of doxycycline (100mg)  -Tablets of artesunate (50mg)  -Tablets of Sulfadoxine pyrimethamine (if no resistance)  -Tablets of acetyl salicylic Acid (500mg)  -Tablets of paracetamol (500mg)  -Bags of ORS  -Bucket with lid and cup (or tap) for handwashing  -Jug of 1 litre with lid to prepare ORS  -NaDCC (0.5mg) tablets to prepare portable water for ORS  -ORS sachets | “1”: ‘Yes’  “0”: ‘No’ |  | |
|  |  |  |  | **Total=35** |  |
|  | Other medical commodities | -Cuff for blood  -Stethoscope  -Thermometer + disinfectant  -Scissors  -Register  -Patients follow up forms  -Broom, floor cloth, dustbin with cover  -Table, chair  -Mats  -Note book for transmission (shifts)  -Pens, permanent marker  -Rubber boots  -Cups, plates, spoons | “1”: ‘Yes’  “0”: ‘No’ |  | |
|  |  | | | **Total=13** |  |
| **IPC Stewardship** | IPC Stewardship | -ORT corners for the treatment of mild/moderate cholera cases  -Separate kitchen for cooking  -Sprayer at entrance, containing a chlorine solution 0.2%  -Sprayers  -Sprayer for insecticide for the whole centre  -20L buckets with cover for chlorine solutions 2% and 0.2%  -Drum 125L for washing hands (containing 0.05% chlorine solution)  -Drum of 125L (0.05% chlorine solution) for hand washing  -Soaps  -Pairs of rubber gloves  -Pierced bed  -Bucket of 10L (separate for stool and for vomit)  -Food store  -Uniforms (one per worker)  -Cooking-pots of 50L  -Showers  -Bucket of 20L with a tap in each shower | “1”: ‘Yes’  “0”: ‘No’ |  |  |
|  |  |  |  |  | |
|  |  | | | **Total=17** |  |
| **Staff training** | Staff training | -Protocol for cholera case management  -Staff training in use of PPE for cholera case management  -Cholera case management guideline pasted on wall | “1”: ‘Yes’  “0”: ‘No’ |  |  |
|  |  | | | **Total=3** | |
