# Supplementary material for: Resource availability and capacity to implement multi-stranded cholera interventions in the north-east region of Nigeria
Source: BMC Glob Public Health. 2023 Aug 4;1:6. doi: 10.1186/s44263-023-00008-3 (PMC11622880; doi:10.1186/s44263-023-00008-3)
Supplement: Supplementary file 4 — Additional file 4. Scores for the coordination of cholera interventions. [file 44263_2023_8_MOESM4_ESM.docx]

**Additional file 4: Scores for the coordination of cholera interventions**

| **Indicator** | **Question** | **Scores for responses** | **Total score** |
| --- | --- | --- | --- |
| **Case management** | -Human resource plan for cholera outbreak response  -Procedure for integrating volunteers into case management during cholera outbreak  -Need assessments determine frequency and content of case management training  -Sufficient resources allocated for case management training  -Supplies for case management periodically tested expired items disposed  -Cholera case management supplies determined on the basis of risk assessments and analyses  -System in place for managing severe cholera cases in community (at scene)  -Standardized cholera triage system in place in the event of a surge  -Guidelines and procedures for the establishment of CTC/CTU/ORP during a cholera outbreak | “1”: ‘Yes’  “0”: ‘No’ | 1 score per question |
|  |  | | **Total=9** |
| **Surveillance** | -TWG leads have access to historical surveillance data to inform planning  -Protocols defining roles/responsibilities/procedures for standardisation  -Surveillance system provide for data-sharing with other cholera stakeholders outside health ministry  -Mechanisms exist for carrying out rapid training health workers for cholera outbreak  -Necessary resources available for training HWs for rapid response to cholera outbreak  -Essential lab supplies and equipment for cholera diagnosis determined on basis of risk assessment  -Essential lab supplies and equipment for cholera diagnosis readily available in sufficient quantity  -Essential lab supplies periodically tested, expired products disposed  -Procedures exist for exceptional procurement of laboratory supplies  -Mechanism for switching from routine cholera surveillance to outbreak surveillance  -Mechanisms to establish a flexible cholera surveillance system that operates in IDP | “1”: ‘Yes’  “0”: ‘No’ | 1 score per question |
|  |  | | **Total=11** |
| **Health system** | -Dedicated cholera TWG that can easily be activated into an EOC during an outbreak  -Does TWG specify the roles and responsibilities of all stakeholders  -Does TWG mobilise and allocate resources for preparedness  -TWG facilitates simulation exercise on cholera outbreak response  -Practice ‘pre-positioning’ to ensure essential supplies available at beginning of an outbreak | “1”: ‘Yes’  “0”: ‘No’ | 1 score per question |
|  |  | | **Total=5** |
| **WASH/OCV** | -TWG work with ministries of environment, water resources, and agriculture  -Mechanisms to ensure adequate WASH services for displaced populations  -Specific roles of OCV stakeholders clearly defined  -OCVs periodically tested, expired vaccines disposed of in line with established guidelines  -System in place, including cold chain, for distribution of OCVs  -Procedures exist for exceptional request and delivery of OCVs for cholera outbreak  -Logistics (e.g., cold chain) in place for implementation of OCV  -Mechanisms to ensure availability OCVs for HWs &vulnerable population (e.g., IDP) | “1”: ‘Yes’  “0”: ‘No’ | 1 score per question |
|  |  | | **Total=8** |
| **Community engagement** | -Responsibilities of pillars/HWs related to cholera risk communication defined  -Reports on cholera TWG published and disseminated regularly  -Risk communication strategies also target minority& vulnerable populations, such as IDPs  -Coordination mechanisms exist to ensure consistent information to the public  -Procedures exist for communicating risk information by community members to public health institutions  -Mechanism for switching from routine to outbreak risk information to community defined | “1”: ‘Yes’  “0”: ‘No’ | 1 score per question |
|  |  | | **Total=6** |
| **Leadership and coordination** | -Regulations guiding entry of foreign/local partners to provide relief services  -Regulations guiding donations of health and medical supplies for cholera response  -Resources and systems (e.g., EOC) sufficient to allow cholera TWG fulfil its mandate  -Funds available for preparedness and response to cholera outbreak  -Procedures for request, acceptance, and utilisation of international financial donations  -Budget for preparedness and response to cholera control/outbreak  -Mechanisms for rapid mobilisation of additional resources to and between state  -Mechanisms for hospital networking during a cholera outbreak  -Logistic system in place that includes tracking, monitoring, and reporting | “1”: ‘Yes’  “0”: ‘No’ | 1 score per question |
|  |  | | **Total=9** |
| TWG= Technical Working Group; EOC= Emergency Operation Centre; IDP=Internally displaced persons; HWs=Healthcare workers | | | |
