# Supplementary material for: Resource availability and capacity to implement multi-stranded cholera interventions in the north-east region of Nigeria
Source: BMC Glob Public Health. 2023 Aug 4;1:6. doi: 10.1186/s44263-023-00008-3 (PMC11622880; doi:10.1186/s44263-023-00008-3)
Supplement: Supplementary file 6 — Additional file 6. Calculation of composite scores for cholera interventions and their indicators. [file 44263_2023_8_MOESM6_ESM.docx]

**Additional file 6: Calculating the composite scores for cholera interventions and their indicators**

The following analysis steps were taken at each of the three stages of analysis identified above:

- We summed the individual scores for responses to each question to determine the total score for a sub-indicator for the cholera case management only. For example, if all the individual criterion under the sub-indicator ‘Clinical staff’ were met, the total score would be ‘3’ as there were three staff categories assessed.
  - Where one sub-indicator matched an indicator (i.e., IPC Stewardship), the sub-indicator total score was used as a numerator (N) in the percentage calculation of the respective cholera case management indicator.
  - Where there was more than one sub-indicator making up an indicator (e.g., Essential staff), an average of the sub-indicators was calculated to obtain the score for the respective indicator that was subsequently used as a numerator (N) in the percentage calculation of the status of indicator implementation.
- In the case of WASH, surveillance and community engagement and coordination, we summed the individual scores for responses to each question to determine the total score for an indicator. For example, if all the individual criteria under the indicator ‘basic hygiene services’ were met, the total score would be '8'.
- In cases where there were multiple indicators measuring one higher level intervention, aggregate indicator scores were added together to obtain an intervention aggregate score which was used as a numerator for calculating the percentage score for that intervention.
- The total possible score for each aggregation level (sub-indicator, indicator, intervention), depending on the response options and coding, ranged between 1 (for example, on a “Yes” and “No” Likert scale) and 3 (on a “on premises”, “up to 500 metres”, and “500 metres or further” Likert scale). The aggregation level total possible score was calculated by adding the total possible scores of its lower aggregation level. The obtained aggregation level total possible score was used as a denominator for percentage score calculation at each level.
- The percentage score for each domain was obtained by dividing the numerator by the denominator (N/D) multiplied by 100.
