# Supplementary material for: Resource availability and capacity to implement multi-stranded cholera interventions in the north-east region of Nigeria
Source: BMC Glob Public Health. 2023 Aug 4;1:6. doi: 10.1186/s44263-023-00008-3 (PMC11622880; doi:10.1186/s44263-023-00008-3)
Supplement: Supplementary file 7 — Additional file 7. Resource availability median scores for cholera interventions and their respective indicators. [file 44263_2023_8_MOESM7_ESM.docx]

**Additional file 7: Resource availability median scores for cholera interventions and their respective indicators**

For all the HCFs in Adamawa and Bauchi States, the resource availability median scores for WASH indicators were good, ranging from high (70-90) to excellent (>90) scores. Notably, resources for ‘basic water services’ (100.0; 85.7-100.0) and ‘basic healthcare waste management services’ (100.0; 75.0-100.0) had an excellent median score in Adamawa State. Meanwhile, in Bauchi State, the resource availability median score for ‘basic healthcare waste management services’ was also excellent at 100.0 (75.0-100.0), while that for ‘basic water services’ was high (85.7; 57.1-100.0). The resource availability median score for ‘basic environmental cleaning practices’ was the lowest among all the WASH indicators in both states (71.4 in Adamawa State and 57.1 in Bauchi State). The resource availability median scores for surveillance indicators were low in general, albeit higher in Adamawa State than in Bauchi State. The resource availability median scores for ‘other medical commodities’ in Adamawa State (69.2; 61.5-76.9) and Bauchi State (61.5; 53.8-76.9) were the highest under case management indicators, seconded by the scores for ‘essential staff’ and ‘supplies for acute rehydration and oral rehydration solution (ORS)’. The resource availability median score for ‘IPC stewardship’ was low across both States (29.4 in Adamawa State and 11.8 in Bauchi State). Notably, the resource availability median score for ‘healthcare personnel’ training was zero in Bauchi State.

| **Resource availability median scores for cholera interventions in healthcare facilities in Adamawa and Bauchi States, Nigeria** | | | | | | | |
| --- | --- | --- | --- | --- | --- | --- | --- |
| **Intervention** | **Indicator** | **Adamawa State**  **(n=55)** | | **Bauchi State**  **(n=65)** | | **Overall**  **(N=120)** | |
|  |  | **Median (IQR)** | **Min-Max** | **Median (IQR)** | **Min-Max** | **Median (IQR)** | **Min-Max** |
| **WASH** | Basic water services | 100.0  (85.7-100.0) | 28.6-100.0 | 85.7  (57.1-100.0) | 0.0-100.0 | 100.0  (71.4-100.0) | 0.0-100.0 |
|  | Basic sanitation services | 80.0  (70.0-90.0) | 10.0-100.0 | 90.0  (70.0-100.0) | 0.0-100.0 | 85.0  (70.0-100.0) | 0.0-100.0 |
|  | Basic hygiene services | 87.5  (62.5-100) | 12.5-100.0 | 87.5  (62.5-100.0) | 25.0-100.0 | 87.5  (62.5-100.0) | 12.5-100.0 |
|  | Basic healthcare waste management services | 100.0  (75.0-100.0) | 0.0-100.0 | 100.0  (75.0-100.0) | 50.0-100.0 | 100.0  (75.0-100.0) | 0.0-100.0 |
|  | Basic environmental cleaning practices | 71.4  (42.9-100.0) | 0.0-100.0 | 57.1  (42.9-71.4) | 0.0-100.0 | 71.4  (42.8-85.7) | 0.0-100.0 |
| **Surveillance and report** | Epidemiology | 44.4  (22.2-77.8) | 11.1-100.0 | 11.1  (0.0-33.3) | 0.0-77.8 | 22.2  (11.1-44.4) | 0.0-100.0 |
|  | Laboratory | 20.0  (0.0-20.0) | 0.0-80.0 | 0.0  (0.0-0.0) | 0.0-80.0 | 0.0  (0.0-20.0) | 0.0-80.0 |
| **Case management** | Essential staff | 66.7  (66.7-100.0) | 33.3-100.0 | 33.3  (33.3-66.7) | 33.3-100.0 | 66.7  (33.3-100.0) | 33.3-100.0 |
|  | Supplies for acute rehydration and ORS | 60.0  (37.1-71.4) | 8.6-88.6 | 28.6  (5.7-48.6) | 0.0-80.0 | 45.7  (20.0-62.9) | 0.0- 88.6 |
|  | Other medical commodities | 69.2  (61.5-76.9) | 38.5-100 | 61.5  (53.8-76.9) | 0.0-100.0 | 69.2  (53.8-76.9) | 0.0-100.0 |
|  | IPC stewardship | 29.4  (17.6-41.2) | 0.0-88.2 | 11.8  (5.9-23.5) | 0.0-52.9 | 17.6  (5.9-29.4) | 0.0-88.2 |
|  | Training of health personnel | 33.3  (0.0-66.7) | 0.0-100.0 | 0.0  (0.0-33.3) | 0.0-100.0 | 33.3  (0.0-66.7) | 0.0-100.0 |
| **Community engagement** | Community engagement | 33.3  (22.2-55.6) | 11.1-77.8 | 22.2  (11.1-44.4) | 0.0-77.8 | 33.3  (11.1-44.4) | 0.0-77.8 |
| Interpretation of availability median scores: low (0-50), moderate (51-70), high (70-90), and excellent (>90) | | | | | | | |
